# Endogenous opioid systems alterations in pain and opioid use disorder

**DOI:** 10.3389/fnsys.2022.1014768

**Published:** 2022-10-19

**Authors:** Jessica A. Higginbotham, Tamara Markovic, Nicolas Massaly, Jose A. Morón

**Affiliations:** ^1^Department of Anesthesiology, Washington University in St. Louis, St. Louis, MO, United States; ^2^Pain Center, Washington University in St. Louis, St. Louis, MO, United States; ^3^School of Medicine, Washington University in St. Louis, St. Louis, MO, United States; ^4^Nash Family Department of Neuroscience and Friedman Brain Institute, Icahn School of Medicine at Mount Sinai, New York, NY, United States; ^5^Department of Neuroscience, Washington University in St. Louis, St. Louis, MO, United States; ^6^Department of Psychiatry, Washington University in St. Louis, St. Louis, MO, United States

**Keywords:** opioids, pain, addicition, opioid use and abuse, opioid use disorder (OUD), reward, endogenous opioids, opioid receptors

## Abstract

Decades of research advances have established a central role for endogenous opioid systems in regulating reward processing, mood, motivation, learning and memory, gastrointestinal function, and pain relief. Endogenous opioid systems are present ubiquitously throughout the central and peripheral nervous system. They are composed of four families, namely the μ (MOPR), κ (KOPR), δ (DOPR), and nociceptin/orphanin FQ (NOPR) opioid receptors systems. These receptors signal through the action of their endogenous opioid peptides β-endorphins, dynorphins, enkephalins, and nociceptins, respectfully, to maintain homeostasis under normal physiological states. Due to their prominent role in pain regulation, exogenous opioids—primarily targeting the MOPR, have been historically used in medicine as analgesics, but their ability to produce euphoric effects also present high risks for abuse. The ability of pain and opioid use to perturb endogenous opioid system function, particularly within the central nervous system, may increase the likelihood of developing opioid use disorder (OUD). Today, the opioid crisis represents a major social, economic, and public health concern. In this review, we summarize the current state of the literature on the function, expression, pharmacology, and regulation of endogenous opioid systems in pain. Additionally, we discuss the adaptations in the endogenous opioid systems upon use of exogenous opioids which contribute to the development of OUD. Finally, we describe the intricate relationship between pain, endogenous opioid systems, and the proclivity for opioid misuse, as well as potential advances in generating safer and more efficient pain therapies.

## Introduction

The intersection between pain and opioid use presents a major dilemma for public health. Efforts to curb the burden of the ongoing opioid crisis continue to be challenged by the need to provide adequate relief for pain patients and at the same time lessen the negative impact of opioid misuse. Pain is extremely prevalent with over half of US adults reporting pain symptoms within the past 3 months (Lucas et al., 2021). Similarly, detriments of opioid abuse are evident in the annual increases in opioid overdose deaths, with the most recent provisional estimates exceeding 80,000 in 2021 (Ahmad et al., 2022). Although the prevalence of problematic opioid use in pain patients is difficult to pin-point for a myriad of reasons (Ballantyne, 2015; Voon et al., 2017), estimates derived from a number of metanalyses suggest rates of problematic prescription opioid use may occur in >80% of pain patients (Minozzi et al., 2013; Ballantyne, 2015; Chou et al., 2015; Vowles et al., 2015; Voon et al., 2017). Collectively, pain and opioid use pose tremendous societal costs, with pain-related health care and lost productivity exceeding $635 billion and opioid abuse-related health care, criminal justice, lost productivity, reduced quality of life, and life lost due to overdose exceeding $1.03 trillion annually (Institute of Medicine Committee on Advancing Pain Research, 2011; Gaskin and Richard, 2012; Florence et al., 2021). Linking the putative relationship between pain and maladaptive opioid use, is the endogenous opioid system, a primary biological substrate of pain and opioid reward. In the present review, we examine how pain and concurrent opioid use may disrupt endogenous opioid system function leading to alterations in reward signaling pathways and ultimately, higher risk for negative outcomes associated with opioid use.

## Problematic opioid use in the context of pain

In 2019, the National Survey on Drug Abuse reported that almost all (>96%) instances of opioid misuse, or use deviating from physicians’ instructions, was restricted to prescription opioid pain medications (Center for Behavioral Health Statistics and Quality, 2019). This same report indicated that among those that misused prescription opioids, the most common reason for misuse was to relieve physical pain (65%). Based on this evidence and the lack of therapeutic alternatives to prescription opioids suggests that the US is undertreating pain or undermining an overlapping and vulnerable population. The former could have likely been fueled by pain management initiatives in the 1990s that recognized pain as a fifth vital sign (Morone and Weiner, 2013; Meisenberg et al., 2018). This notion encouraged physicians to prioritize pain reduction through the liberalization of opioid prescriptions (Compton and Volkow, 2006; U.S. Department of Health and Human Services, 2019) which led to the initial wave of prescription overdose deaths (Rudd et al., 2016). This was addressed by several opioid diversion and mitigation strategies, including revisions to opioid prescribing practices in 2016 by the Center of Disease Control (CDC) that limit the number of opioid prescriptions (Lappin, 2016; Volkow and McLellan, 2016). Although these efforts appeared to bring prescription overdoses to a plateau, synthetic opioid overdoses (both illicit and prescribed) increased at alarming rates (CDC WONDER, 2018). It is difficult to pin down whether the continued rise in opioid overdoses was driven by the unmet needs of pain patients, growth in illicit markets, or a combination of both. Despite additional government-backed initiatives intended to curb opioid use and facilitate research for pain management alternatives (U.S. Department of Health and Human Services, 2019), the prevalence of chronic pain and opioid overdose deaths continue to rise each year (Goldstick et al., 2021; Zajacova et al., 2021; Ahmad et al., 2022), and have even been amplified by the COVID-19 pandemic (Fallon et al., 2021; Manchikanti et al., 2021; Soares et al., 2021). The National Institute of Health’s (NIH) most recent endeavor, the HEAL initiative (Helping to End Addiction Long-term), recognized the need to address the opioid crisis through improvements to pain management (Wandner et al., 2022). As such, our ability to curtail opioid abuse and improve the treatment of pain relies heavily on our capacity to understand the neurobiological mechanisms underlying pain and opioid systems.

According to the International Association for the Study of Pain (IASP), pain is defined as “an unpleasant sensory and emotional experience associated with, or resembling that associated with, actual or potential tissue damage” (Raja et al., 2020). The intersection between these two dimensions of pain—sensation and emotion—present a substantial problem for chronic pain patients on long-term opioid therapy which can play a synergistic role in perpetuating pain, mood disruptions, and problematic opioid use. The occurrence of mood disorders can predict not only opioid misuse liability (NIDA, 2008; Davis et al., 2017; Center for Behavioral Health Statistics and Quality, 2019; Jones and McCance-Katz, 2019; Smit et al., 2020) but also, susceptibility to pain conditions (Viana et al., 2018; Rizvi et al., 2021). Likewise, patients with opioid use disorder (OUD), a chronic and relapsing disorder characterized by persistent and compulsive drug-seeking behavior despite negative outcomes, frequently report comorbidities of chronic pain (up to 65%) and mood disorders (up to 82%) (Davis et al., 2017; Hser et al., 2017; Peciña et al., 2018; Jones and McCance-Katz, 2019; Higgins et al., 2020; Latif et al., 2021). It is therefore not surprising that chronic pain patients are 2–3 times more likely to meet diagnostic criteria for an anxiety, mood, and mental disorders (Pereira et al., 2017) and are at higher risk (>50%) for developing opioid or substance use disorder (Højsted and Sjøgren, 2007; Morasco et al., 2011). Collectively, the co-occurrence of pain, mood disruptions, and problematic opioid use can have additive effects on the severity and risk for the other. The extensive overlap of these conditions alludes to a common underlying mechanism; and while each of these conditions are associated with dysfunction across multiple biological systems, one potential source of shared functional disruption lies within the endogenous opioid system (Jarcho et al., 2012; Witkin et al., 2014; Peciña et al., 2018; Jones and McCance-Katz, 2019; Toubia and Khalife, 2019).

## The endogenous opioid system

Both pain and exogenous opioids can disrupt function of the endogenous opioid system (Roeckel et al., 2016) and similarly, alterations in endogenous opioid activity can predict variations in pain thresholds, opioid-induced analgesia, and the proclivity for opioid misuse and abuse (Corder et al., 2018; Jassar et al., 2019; Llorca-Torralba et al., 2019a; Massaly and Morón, 2019; Bodnar, 2021). The endogenous opioid system plays an important role in analgesia, but it is also critically involved in autonomic regulation, immunological responses, gastrointestinal function, learning and memory, and many other functions (Bodnar, 2021). As such, the endogenous opioid system is crucial for maintaining homeostasis and alterations in its activity are largely state dependent (Darcq and Kieffer, 2018; Valentino and Volkow, 2018). This system is also highly integrated with other biological systems involved in stress regulation, mood, and reward such as the endocannabinoid, serotonin, oxytocin, vasopressin, and dopamine (DA) systems and the hypothalamic adrenal pituitary axis (Leknes and Tracey, 2008; Toubia and Khalife, 2019; Emery and Akil, 2020; Koob, 2020; Bodnar, 2021; Mohammadkhani and Borgland, 2022). Implicitly, the extensive crosstalk between these contributes to the highly adaptive nature of the opioid system and its ability to acutely respond to noxious stimuli. However, chronic perturbations to opioid systems can leave the system vulnerable to dysfunction and have debilitating consequences (Stoeber et al., 2018). Here, we focus on the impact of pain and opioids on function of the endogenous opioid system and reward pathways and examine their putative role in provoking maladaptive patterns of opioid use and OUD.

### Opioids

Opioids are natural, synthetic, or semi-synthetic chemicals acting on opioid receptors to produce analgesia among other peripheral effects (Zöllner and Stein, 2007). Opium is a dried milky exudate obtained from the unripe seed pods of the opium poppy, *papaver somniferum* (Brownstein, 1993). Among the dozens of alkaloids found in opium, the pharmacologically relevant constituents include morphine (10–15%), codeine (1–3%, noscapine (4–8%), papaverine (1–3%), and thebaine (1–2%) (Zöllner and Stein, 2007). The antiquity of opium for medicinal use was documented as early as ∼2100 BCE in Sumerian medical tablets (Duarte, 2005). The unrivaled ability of opium to relieve pain was recognized in texts for millennia, but the therapeutic application of opioids was transformed when a young German apothecary’s assistant, F.W.A. Sterürner, isolated crystalline morphine (1803–1817), naming it after Greek god of sleep and dreams (Krishnamurti and Rao, 2016). The subsequent invention of the hypodermic syringe needle in the 1850s facilitated the use of morphine for surgical procedures, pain relief, and as an adjunct to general anesthetics (Brownstein, 1993). Since then, the broad application of various opioid analgesics has facilitated a greater understanding of the opioid system and the clinical utility of opioids for pain management.

The existence of opioid receptors was first proposed in the 1950s (Beckett and Casy, 1954), but it was not until the 1970s that different bioassays began to identify stereospecific binding sites for opioids in the brain (Pert and Snyder, 1973; Simon et al., 1973; Terenius, 1973; Martin et al., 1976; Lord et al., 1977). These studies revealed that exogenous opioid ligands produce their narcotic effects through actions at different opioid receptors which led to the discovery that endogenous opioid-like peptides can produce similar effects through their activity at the same peptide receptors (Cox et al., 1976; Hans et al., 1977; Olson et al., 1979). The first evidence of distinct opioid receptor types was determined by detailing the actions of several analgesic drugs. As such, the first two opioid receptor types were named after the prototypic drugs used in these studies to distinguish them, mu (μ) for morphine and kappa (κ) for ketocyclazocine (Martin et al., 1976). Pharmacological analysis revealed a third opioid receptor type in the mouse vas deferens that exhibited a pharmacological profile markedly different from those previously identified (μ and κ) and was accordingly, named delta (δ) to signify this difference (Lord et al., 1977). The heterogeneity of these receptor types was later confirmed when distinct mRNAs for each receptor type were cloned and characterized (Evans et al., 1992; Kieffer et al., 1992; Chen et al., 1993; Yasuda et al., 1993). Together, the μ, κ, and δ opioid receptors (MOPR, KOPR, DOPR, respectively) are considered the classical opioid peptide receptors based on their structural homology and sensitivity to the non-selective opioid receptor antagonist, naloxone (Dietis et al., 2011). A fourth opioid receptor-like (OPRL1) gene was later revealed to encode a receptor with a primary structure analogous to previously identified opioid receptors and yet, it lacked sensitivity to traditional opioid ligands (Bunzow et al., 1994; Mollereau et al., 1994). As such, OPRL1 remained an ‘orphan’ receptor until two independent groups isolated its endogenous ligand, nociception (Meunier et al., 1995) or orphanin FQ (Reinscheid et al., 1995) (N/OFQ), for which the OPRL1 or N/OFQ opioid receptor is referred to here on as NOPR. While MOPR, KOPR, DOPR, and NOPR comprise the four major opioid receptor systems due to homology in structure and function, NOPR is often excluded from “classical” opioid receptor types based on its lack of sensitivity to naloxone or prototypical opioid ligands.

Four major opioid peptide families are commonly associated with complimentary opioid receptor systems for which they exhibit preferential activity: β-endorphins (MOPR), dynorphins (KOPR), enkephalins (DOPR), and N/OFQ (NOPR) (Hughes et al., 1977; Nakanishi et al., 1979; Horikawa et al., 1983; Pathan and Williams, 2012; Shenoy and Lui, 2018). However, despite greater selectivity of these endogenous ligands and their respective receptors, the activity of both endogenous and exogenous opioids at distinct receptor types is rarely exclusive to one family and can often activate multiple receptor types to varying degrees (Stein, 2016). Opioid receptors are seven-transmembrane G protein-coupled receptors (GPCR) that generally couple to inhibitory G proteins, thereby reducing signal transduction and neurotransmission by engaging several second- and third-messenger systems and regulating ion channel activity (Zöllner and Stein, 2007; Al-Hasani and Bruchas, 2011; Toll et al., 2016; Corder et al., 2018). Different opioids can also engage biased signaling pathways to preferentially activate GPCR-dependent signaling or β-arrestin-dependent signaling, which can produce analgesia or unwanted side-effects, respectively (Ballantyne and Chavkin, 2020). Allosteric binding sites on opioid receptors, distinct from orthostatic sites or the ligand binding pocket, can also modulate opioid receptor function through activation by various other neurotransmitters and neuropeptides (Kathmann et al., 2006; Burford et al., 2015; Remesic et al., 2017; Livingston and Traynor, 2018). For example, cannabidiol (CBD), an exogenous cannabinoid ligand, can act as a negative allosteric modulator at MOPR or DOPR in rat cerebral cortices to reduce their function (Kathmann et al., 2006). Positive allosteric modulators for MOPR have also been sought after as they may reduce some of the unwanted side-effects attributed to traditional opioid medications or facilitate the activity of endogenous opioids (Burford et al., 2015). Adding another layer of complexity to opioid receptor signaling is the fact that different opioid receptors can associate with each other to form heteromers (e.g., MOPR-DOPR, DOPR-KOPR, KOPR-MOPR). For example, DOPR antagonism of DOPR-MOPR heteromers can act to enhance MOPR agonist-mediated analgesia (Gomes et al., 2004). The complexity of opioid receptor signaling mechanisms shed light on the multiple means by which opioid system function can be disrupted.

Opioid receptors are among the most widely expressed receptors in the central and peripheral nervous systems, although the composition and distribution of different opioid receptor types varies across regions (Corder et al., 2018). In the periphery, opioid receptors expressed in the lungs, heart, kidney, small intestine, and pancreas, can modulate organ function, inflammation, as well as multiple homeostatic processes (Peng et al., 2012). Opioid receptors can also be found in neuroendocrine (adrenals, pituitary), immune (leukocytes), and ectodermal cells, where they can modulate nociception and inflammation (Zöllner and Stein, 2007; Stein, 2013). In the context of pain, opioid receptors are ideally situated among, and connected with, somatosensory neurons of dorsal root ganglion (DRG) and second-order neurons of the dorsal horn of the spinal cord where they transmit ascending nociceptive signals to cortical areas through the spinothalamic, spinoreticular, and spinoparabrachial pathways (Basbaum and Fields, 1984; Marchand, 2008; DosSantos et al., 2017; Ringkamp et al., 2018). Local release of endogenous opioids or acute application of exogenous opioids at injury sites can suppress DRG activity to reduce nociceptive signaling and pain perception (Dickenson et al., 1990; Stein et al., 2003; Spahn et al., 2017; Corder et al., 2018; Massaly et al., 2020). Similarly, top-down regulation by opioid receptor systems within the periaqueductal gray (PAG) and rostral ventral medulla (RVM) can exert descending modulatory control over nociceptive signal transduction (Marchand, 2008; Ringkamp et al., 2018). The level of top-down control over anti-nociceptive responses can also be influenced by opioid receptor systems in other brain regions involved in cognition, affect, sensation, and motivation (Corder et al., 2018; Bannister and Dickenson, 2020; Dickenson et al., 2020). As such, the central and peripheral presence of opioid systems yields the ability of opioid receptors to functionally modulate reward-aversion networks through ascending and descending modes of control, and therefore, play a substantial role in aversive pain states, reward from pain relief, and hedonic balance (Darcq and Kieffer, 2018).

Proper functioning of the endogenous opioid system is essential for survival mechanisms involved in reward- and aversion-based learning and behavior. When the integrity of this system becomes compromised, the ability to integrate opioid reward- and pain aversion-related information will also become impaired. Among the many debilitating consequences associated with compromised opioid system function, is the risk of OUD. After repeated drug exposure, reward-processing centers can undergo neuroadaptations that leave affected individuals with enhanced incentive salience and habit formation, impulsivity, stress reactivity, and negative affect in the absence of drug; thereby producing overall disruptions in motivation (Koob and Volkow, 2010). As a result, maladaptive drug use is perpetuated through cycles of binge/intoxication, withdrawal/negative affect, and preoccupation/craving (Koob and Volkow, 2010). OUD and other substance use disorders are linked with adaptations to the opioid system (Darcq and Kieffer, 2018) because of its central role in reward processing (le Merrer et al., 2009). Therefore, the ability of the opioid system to regulate both pain states and the actions of opioid drugs may exacerbate the risk for the development of OUD in pain patients on long-term opioid therapies. Here, we focus on adaptations within mesolimbic reward pathways and the putative synergistic effects of pain and opioid use in driving opioid misuse liability.

### μ Opioid receptor

The role of MOPR in mediating opioid-dependent analgesia and reward provides support for the abundance of research on this opioid receptor family. The analgesic effects of MOPR activity are attributed to their hyperpolarizing effects and suppression of neuronal activity. This is regulated by G_αi_-mediated inhibition of cAMP production (Raffa et al., 1994), activation of G protein-coupled inwardly rectifying potassium (GIRK) channels (Ikeda et al., 2000), G_βγ_ -mediated inhibition of L-type calcium channels (Bourinet et al., 1996), and inhibition of voltage-dependent calcium channels (VDCC) (Saegusa et al., 2000). Alternatively, β-arrestins can modulate MOPR signaling by decoupling the receptor from G proteins and facilitating receptor internalization (Siuda et al., 2017; Cong et al., 2021). β-arrestins can also engage multiple intracellular signaling cascades independent of G proteins (Macey et al., 2006) and biased signaling mechanisms through β-arrestins or G proteins often produce distinct effects (discussed below).

Endogenous MOPR agonists, like β-endorphins, can be locally released at injury sites to provide acute pain relief through their signaling at the MOPR (Hassan et al., 1993; Truong et al., 2003; Stein et al., 2009). Similarly, acute administration of exogenous MOPR agonists, like morphine, can provide both pain relief and reinforcement. Evidence from positron emission tomography (PET) studies in humans demonstrate that acutely painful stimuli increase MOPR activity in multiple brain regions, including those implicated in nociception and reward processing, such as the PAG and the nucleus accumbens (NAc), respectively (Zubieta et al., 2001, 2002; Bencherif et al., 2002). Relative to pain-free conditions, acute pain enhances MOPR activity while its activity is decreased under conditions of chronic pain. In animal models of neuropathic pain, MOPR expression is downregulated in the spinal cord, DRG, and several cortical regions in the days and weeks following injury (Porreca et al., 1998; Zhang et al., 1998; Rashid et al., 2004; Pol et al., 2006; Thompson et al., 2018). Similarly, patients with chronic lower back pain exhibit lower circulating levels of β-endorphin (Bruehl et al., 2012, 2014, 2017, 2013; Rhodin et al., 2013), while deficits in MOPR binding potential have been linked with multiple pain conditions including fibromyalgia, chronic migraine, trigeminal neuropathic pain, and chronic lower back pain (Harris et al., 2007; DosSantos et al., 2012; Hagelberg et al., 2012; Martikainen et al., 2013; Schrepf et al., 2016; Jassar et al., 2019; Toubia and Khalife, 2019). Therefore, the function of the MOPR system can differ depending on the persistence of pain conditions, losing efficacy over time.

Importantly, MOPR activity can contribute to both sensational and emotional aspects of pain. In healthy controls, baseline MOPR binding can predict pain thresholds, such that lower MOPR binding in multiple cortical regions is associated with higher pain sensitivity (Zubieta et al., 2001, 2002; Hagelberg et al., 2012). Moreover, MOPR binding is negatively correlated with affective pain ratings (Zubieta et al., 2001, 2002), adding further support to the idea that MOPR activity can modulate sensory and affective components of pain. In patients with various chronic pain conditions, the ability of MOPR binding to predict pain sensitivity is similar. For example, patients with trigeminal neuropathic pain exhibit reduced MOPR binding in the NAc which is negatively correlated with pain ratings (DosSantos et al., 2012). Consistent with this relationship, reduced MOPR binding in the prefrontal cortex is associated with migraine severity (DaSilva et al., 2014). Similar results have been recapitulated in rodent models of neuropathic pain. Months after spared nerve injury, rats show reduced MOPR availability and expression in the insula, caudate putamen, and motor cortices, and these levels are correlated with deficits in sucrose preference, a measure of anhedonia (Thompson et al., 2018). Together, these findings indicate that chronic pain disrupts MOPR function to negatively regulate sensory and affective components of pain.

The MOPR system is also influenced by acute or chronic exposure to exogenous opioids. In patients undergoing surgery under general anesthesia, plasma β-endorphin levels are increased, and this effect is inhibited by administration of fentanyl, a potent MOPR agonist (Dubois et al., 1982; Cork et al., 1985). Fentanyl administration also induces MOPR phosphorylation in the striatum of mice at sites involved in receptor desensitization and internalization (Macey et al., 2006), suggesting that acute opioid exposure can have rapid effects on receptor desensitization and tolerance. In contrast, MOPR antagonism increases β-endorphin levels (Hargreaves et al., 1986), adding further support to the idea that endogenous β-endorphin release is regulated by MOPR activity. Chronic opioid exposure can have detrimental effects on endogenous opioid production and MOPR system function. For example, chronic morphine treatment reduces expression levels of the β-endorphin precursor protein, proopiomelanocortin (POMC), in rats (Bronstein et al., 1990; Wardlaw et al., 1996; Przewlocki, 2004), and reduces MOPR density in β-endorphin-expressing neurons of the hypothalamus (site of synthesis) in guinea pigs (Zhang et al., 1996). As such, chronic exposure to exogenous MOPR agonists reduce MOPR system function by reducing endogenous production of MOPR agonists (β-endorphins) and overall MOPR availability. Chronic opioid exposure can also alter function of remaining MOPR by producing a switch in MOPR G-protein coupling from Gi/o to Gs, leading to activation of adenylyl cyclase rather than inhibition (Wang et al., 2005). MOPR activation and subsequent phosphorylation by GPCR kinases can also lead to the recruitment of β-arrestins, which—in conjunction with many other effectors—leads to MOPR receptor desensitization and internalization (Koch and Höllt, 2008; Roeckel et al., 2016; Corder et al., 2017; Derouiche et al., 2020; Massaly et al., 2021). MOPR phosphorylation at sites involved in receptor desensitization and internalization are observed in mice seven days after partial sciatic nerve ligation, a manipulation that produces tolerance to both the analgesic and conditioned reinforcing properties of morphine (Petraschka et al., 2007). Together, these disruptions to endogenous opioid production and MOPR function in response to chronic opioid exposure can lead to long-term plasticity underlying the development of opioid-induced hyperalgesia, analgesic tolerance, and negative affect, contributing to problematic opioid use.

The ability of pain and exogenous opioids to modify MOPR system function can lead to alterations within the mesolimbic reward pathway that may “prime” the system to be more vulnerable to the abuse of opioids, alcohol, and other substances of abuse (Contet et al., 2004). Opioid activity at MOPR produces rewarding effects by hyperpolarizing GABAergic inputs onto ventral tegmental area (VTA) DA neurons, thereby disinhibiting DA release (Elman and Borsook, 2016; Mitsi and Zachariou, 2016; Stoeber et al., 2018). Local infusion of MOPR agonists in the VTA is sufficient to produce reinforcing behaviors and conditioned reward-seeking behavior (Devine and Wise, 1994). Additionally, VTA MOPR function is necessary for opioid-dependent reward (Cui et al., 2014). Based on the ability of opioids to provide both positive reinforcement and pain relief, it seems evident that pain-induced alterations on MOPR signaling within mesolimbic circuits may facilitate tendencies toward opioid abuse (Koob, 2020). A large body of evidence indicates that pain augments opioid reward thresholds by disrupting DA transmission within the mesolimbic system (Hipólito et al., 2015; Martikainen et al., 2015; Taylor et al., 2016; Selley et al., 2020; Ren et al., 2021). This is regulated at least partly by deficits in MOPR system function (Markovic et al., 2021). Preclinical studies have shown that inflammatory and nerve injury pain reduces MOPR agonist efficiency at silencing VTA GABAergic transmission (Hipólito et al., 2015; Taylor et al., 2015), thus decreasing the ability of MOPR agonists to disinhibit VTA DA neurons (Ozaki et al., 2004, 2003, 2002; Hipólito et al., 2015) and evoke DA release in the nucleus accumbens (NAc) (Niikura et al., 2010; Hipólito et al., 2015; Taylor et al., 2015). These pain-induced deficits in mesolimbic function significantly dampen the rewarding properties of MOPR agonists. For example, rats with sciatic nerve ligation exhibit reduced placed preference induced by intra-VTA administration of the MOPR agonist, DAMGO, or systemic administration of morphine—an effect paralleled by attenuated MOPR binding in the VTA (Niikura et al., 2008). Consistent with this idea, chronic pain patients at low risk for opioid misuse exhibit less pain-induced activation of MOPR in the NAc, and this effect is associated with fewer mood disturbances and negative affect (Ballester et al., 2022). Taken together, MOPR signaling is a primary mechanism by which opioids yield high potential for abuse. As such, the MOPR system has received interest as therapeutic target for the treatment of chronic pain and OUD since the 1960s. Methadone, a long-acting MOPR agonist, has been used as a substitution therapy for chronic pain patients with long-term opioid therapy and maintenance treatment for patients with OUD (Kreek, 1973, 1991, 2000; Ferrari et al., 2004; Axelrod and Reville, 2007; Shi et al., 2008; Mattick et al., 2009; Kreek et al., 2010). The unique pharmacokinetic profile of methadone (slow onset, slow offset) yields a useful strategy to target the MOPR system while reducing the potential for opioid abuse, but the efficacy of these treatments is often limited by inter-individual variability, resources, and appropriate implementation (Dole and Nyswander, 1976; Ward et al., 2009; Kreek et al., 2010). As such, recent approaches have examined allosteric modulators of MOPR and biased signaling mechanisms as a means of offsetting the negative side effects of opioid pain medications (Manglik et al., 2016). A better understanding of how different pain conditions alter MOPR function with consideration of the interplay with ongoing opioid use will aid the development of future pharmacotherapeutic targeting strategies.

### κ Opioid receptor

In contrast to the rewarding effects exerted by MOPR activity, the KOPR system is often attributed to dysphoria, anhedonia, and aversion (Spanagel et al., 1992; Darcq and Kieffer, 2018; Liu et al., 2019; Massaly et al., 2019; Cahill et al., 2022b). The opioid peptide, dynorphin, and its activity at KOPR have been implicated in negative affect, pain, analgesia, stress, and addiction (Bruchas et al., 2009; Darcq and Kieffer, 2018). A large body of evidence demonstrates that pain increases dynorphin mRNA expression and peptide production in the spinal cord of rodents and humans (Iadarola et al., 1988; Millan et al., 1988, 1985; Samuelsonn et al., 1993; Xu et al., 2004; Podvin et al., 2016; Liu et al., 2019). Following the onset of pain, the increase in dynorphin parallels the development of hyperalgesia and KOPR antagonism can facilitate hyperalgesic responses (Millan et al., 1987; Xu et al., 2004). This suggests that the dynorphin-kappa system is actively recruited under pain conditions to suppress nociceptive transmission. However, the ability of KOPR activity to suppress hyperalgesic responses may be dependent on the cell populations activated by dynorphin. For example, spinally restricted dynorphin signaling at KOPR expressed in astrocytes, rather than neurons, can produce nociceptive responses (Chartoff and Mavrikaki, 2015; Cahill et al., 2022b). In this regard, astrocytic KOPR activation can trigger hypertrophy in spinal astrocytes to facilitate the persistence of pain and the development of MOPR analgesic tolerance (Donnelly et al., 2020).

Pain can also trigger dynorphin-mediated KOPR activity in supraspinal regions. Pain induced adaptations to KOPR function within mesolimbic pathways may represent a primary mechanism by which pain can lead to the emergence of negative affect and altered motivational states. Indeed, pain conditions increase dynorphin expression and KOPR activity in multiple supraspinal sites including the VTA and NAc (Narita et al., 2005; Tejeda et al., 2017; Liu et al., 2019; Massaly et al., 2019; Navratilova et al., 2019; Wawrzczak-Bargieła et al., 2020). Although genetic deletion of KOPR or KOPR antagonism fails to alter pain-induced hyperalgesia, these manipulations can effectively restore pain-induced anhedonia and aversion (Narita et al., 2005; Tejeda et al., 2017; Liu et al., 2019; Massaly et al., 2019; Navratilova et al., 2019; Vergara et al., 2020). Recent evidence suggests that KOPR activity in NAc may be important for the transition from acute to chronic pain. Using hind paw injections of prostaglandin E2 to induce a persistent hyperalgesic state in rats, KOPR manipulations did not affect mechanical sensitivity during the induction phase (14 daily injections) (Vergara et al., 2020). Rather, intra-NAc KOPR agonists or antagonists facilitated or inhibited the persistence of hyperalgesia, respectively (Vergara et al., 2020). The findings suggest that the KOPR system may play an important role in pain chronification (Borsook et al., 2016).

Dynorphin recruitment under conditions of pain and the ability of KOPR activity to drive the transition from acute to chronic pain, suggest that KOPR may also be important for the development of comorbidities associated with persistent pain states such as negative affect and motivational deficits (Al-Hasani et al., 2015; Hipólito et al., 2015; Taylor et al., 2015; Elman and Borsook, 2016; Liu et al., 2019; Massaly et al., 2019). In general, KOPR agonists produce aversion and are associated with negative affect across species. In humans, KOPR agonists have psychotomimetic effects and produce dysphoria and hallucinations (Pfeiffer et al., 1986; Ranganathan et al., 2012) while increasing circulating stress hormone levels of cortisol (Ur et al., 1997). Similarly, in rodent models, both systemic and intracranial injections of KOPR agonists are sufficient to produce a conditioned place aversion (CPA) (Chefer and Ba, 2013; Tejeda et al., 2013) and increases in circulating levels of the stress hormone, corticosterone (Hayes and Stewart, 1985; Iyengar et al., 1986). These findings indicate that dynorphin-mediated activation of KOPR is acutely aversive and stimulates HPA axis activity, a putative mechanism contributing to negative affect associated with pain conditions. In support of this, increases in NAc dynorphin are found in suicidal individuals (Hurd et al., 1997) and animal models of depression (Carlezon and Krystal, 2016; Tejeda and Bonci, 2019). Importantly, these effects appear to be driven by the ability of KOPR activity to attenuate DA release in the NAc (Chefer and Ba, 2013; Conway et al., 2019; Escobar et al., 2020).

Dynorphin recruitment in mesolimbic pathways under conditions of pain leads to motivational deficits. For example, our lab showed that inflammatory pain increases KOPR function and recruits dynorphin-containing neurons in the NAc shell (Massaly et al., 2019). In this work, we found that the recruitment of NAc shell dynorphin neurons and activity at KOPR is both necessary and sufficient to drive pain-induced motivational deficits for natural rewards (Massaly et al., 2019). These effects also translate to motivational deficits for opioid drug reward. In models of neuropathic or inflammatory pain, morphine-induced conditioned place preference (CPP) scores are attenuated but can be restored by intra-NAc infusions of KOPR antagonists (Narita et al., 2005; Liu et al., 2019). Moreover, pain reduced opioid-evoked DA release in the NAc, an effect restored by intra-systemic KOPR antagonism (Narita et al., 2005; Liu et al., 2019). This suggests that pain-induced recruitment of dynorphin significantly decreases opioid reward processing. Importantly, KOPR antagonism does not impact opioid reward or dopamine release in the absence of pain (Liu et al., 2019), further implicating the state-dependent role of dynorphin. Opioid exposure, in the absence of pain, can perturb KOPR function in a manner similar to pain. For example, opioid self-administration or chronic opioid exposure increases prodynorphin (dynorphin precursor) levels in the NAc (Nylander et al., 1995; Trujillo et al., 1995; Solecki et al., 2009; Schlosburg et al., 2013). Based on this, pain patients on long-term opioid therapies may have compounding effects of pain and opioid use on KOPR dysfunction, exacerbating motivational deficits, negative affect, and leading to increased risk for maladaptive opioid use. Consistent with this idea, genetic polymorphisms to the prodynorphin gene have been linked with increased risk for OUD (Clarke et al., 2012).

The role of the KOPR system in pain-related mood disturbances and negative affect make this system an appealing target from a treatment perspective (Roeckel et al., 2016; Jassar et al., 2019; Llorca-Torralba et al., 2019b). Although systemic KOPR agonists can produce analgesia, many undesirable effects including hallucinations, impaired stress-coping skills, and deficits in reward-driven motivation, limit their clinical utility as therapeutic alternatives to traditional exogenous opioids (Jarcho et al., 2012; Davis et al., 2017; Jones and McCance-Katz, 2019; Toubia and Khalife, 2019; Emery and Akil, 2020). However, pharmacotherapies with partial agonist properties at KOPR have been examined in clinical trials for treatment of alcohol use disorder (AUD). Nalmefene, a MOPR inverse agonist and weak partial KOPR agonist can effectively reduce alcohol consumption and heavy drinking days (Barrio et al., 2018; Miyata et al., 2019), while improving emotional processing in AUD patients (Vollstädt-Klein et al., 2019). On the other hand, considering the upregulated KOPR signaling in supraspinal sites driving negative affective states under pain conditions, the development of KOPR antagonists may yield promising therapeutic potential for the treatment or prevention of neuropsychiatric disorders comorbid with pain (Ghozland et al., 2002; Liu et al., 2019; Escobar et al., 2020; Ji et al., 2021; Cahill et al., 2022b). Buprenorphine is a KOPR antagonist/partial agonist, a partial MOPR and NOPR agonist, and DOPR antagonist with higher efficacy in the periphery than centrally (Bloms-Funke et al., 2000; Lutfy and Cowan, 2004). As such, this treatment provides higher levels of analgesia while sparing many of the negative side-effects associated with traditional opioid medications (Cowan et al., 1977; Lutfy and Cowan, 2004; Koppert et al., 2005; Gudin and Fudin, 2020). The ability of buprenorphine to reduce depressive symptoms has been demonstrated in patients with treatment resistant depression (Karp et al., 2014) and patients with comorbid depression and OUD (Yovell et al., 2016; Ahmadi et al., 2018a). Adding further support to this strategy, buprenorphine is also effective in reducing pain severity in experimentally-induced pain (Koppert et al., 2005) and pain patients (Pergolizzi and Raffa, 2019; Gudin and Fudin, 2020). In patients with OUD and pain symptoms, combinatorial therapeutic approaches with buprenorphine and naloxone can effectively reduce pain severity (Worley et al., 2017, 2015; Shulman et al., 2020). The ability of similar strategies to curb opioid use and craving are less consistent (Blondell et al., 2010; Ahmadi et al., 2018b; Parida et al., 2019). However, evidence suggests that the efficacy of buprenorphine as a substitution therapy for OUD is dependent on the dose and rate of tapering (Walsh et al., 1994; Sturgeon et al., 2020), but concerns remain for the potential for abuse (Cicero et al., 2018). To advance KOPR targeting strategies, it will be critical for future research to dissociate the analgesic properties of spinal KOPR and the emotional component of pain mediated by supraspinal KOPR. Biased ligands and peripherally restricted pharmacotherapeutics targeting KOPR will be important developments for treating the mood disruptions in the context of pain.

### δ Opioid receptor

The DOPR system plays an important role in pain, analgesia, and negative affective states (Quirion et al., 2020). Similar to KOPR, the functional role of the DOPR system may be selectively dependent on pain states. In rodent models of inflammatory or neuropathic pain, DOPR expression increases in the dorsal horn of the spinal cord and DRG neurons (Cahill et al., 2003; Morinville et al., 2004a; Kabli and Cahill, 2007). The recruitment of DOPR in pain conditions appears to have an inhibitory influence over nociception because genetic deletion of DOPR, but not MOPR, exacerbates and prolongs thermal and mechanical sensitivity in mice with inflammatory pain (Gavériaux-Ruff et al., 2008). Similarly, conditional knock-out of DOPR in the peripheral nociceptive neurons exacerbates mechanical sensitivity in conditions of inflammatory or neuropathic pain (Gaveriaux-Ruff et al., 2011). Moreover, systemic, or local DOPR agonism effectively reduces mechanical and thermal hyperalgesia in wild-type, but not DOPR knock-out, mice, adding further support to the anti-nociceptive role of DOPR (Gaveriaux-Ruff et al., 2011). Importantly, the role of DOPR in nociception is dependent on the presence of pain. In the absence of pain, DOPR activity has negligible effects on analgesia; but in the presence of neuropathic or inflammatory pain, DOPR agonists can reduce thermal and mechanical pain sensitivity (Cahill et al., 2001; Gendron et al., 2007a,b; Normandin et al., 2013). DOPR agonists have also been shown to attenuate migraine associated-pain in preclinical models *via* signaling through calcitonin gene-related peptide (Moye et al., 2021). The weak antinociceptive effects of DOPR agonists in pain naïve animals results from low levels of DOPR expression in plasma membrane. In conditions of pain, the density of DOPR increases at the membrane and cell surface in spinal cord regions and DRG neurons (Quirion et al., 2020). The ability of pain to increase DOPR trafficking is a potential cellular mechanism to explain the pain selective analgesic properties of DOPR agonists. DOPR trafficking is controlled by constitutive pathways involving dynamic remodeling of actin filaments of the cytoskeleton (Mittal et al., 2013) or regulated signaling pathways involving G-protein receptor kinases (GRKs) (Quirion et al., 2020), but the precise mechanisms of DOPR trafficking remain unclear. The DOPR system can modulate nociceptive components of pain not only through neuronal mechanisms, but astrocytic mechanisms as well. For example, deletion of astrocytic DOPR decreases cold allodynia in neuropathic pain while mechanical allodynia is not affected (Reiss et al., 2021). In contrast, DOPR activity in somatostatin-expressing neurons of the dorsal horn of the spinal cord can reduce mechanical, but not thermal, sensitivity in neuropathic pain models (Wang et al., 2018). Therefore, DOPR can modulate distinct elements of the nociceptive experience based on their activity in different cellular populations.

The DOPR system has also received a lot of attention for its role in emotional regulation of mood disorders like anxiety and depression. For example, genetic ablation of DOPR or DOPR antagonists has anxiogenic effects in animal models, while DOPR agonists produce opposite effects (Filliol et al., 2000; Saitoh et al., 2005; Narita et al., 2006a,b; Perrine et al., 2006; Bilkei-Gorzo et al., 2007; Chu Sin Chung and Kieffer, 2013). Similarly, DOPR agonists are associated with higher latency for immobility in the forced swim task, a measure of depressive-like behavior in rodent models (Filliol et al., 2000; Jutkiewicz et al., 2006; Torregrossa et al., 2006), suggesting that pain-related recruitment of DOPR may function to offset mood dysregulation in pain. Unlike MOPR, DOPR activity is not rewarding in the absence of pain. DOPR agonists can elicit CPP in mice with peripheral nerve injury, but not sham controls, while DOPR antagonists selectively produce CPA in mice with pain (Cahill et al., 2022a). This demonstrates the pain state-dependent role of DOPR and suggests that DOPR activation acts as negative reinforcer by alleviating pain rather than producing positive reinforcement.

Given the ability of the DOPR system to modulate analgesic responses and negative affect while sparing any properties that may lead to abuse, DOPR have been investigated for their potential role in curbing opioid use (Quirion et al., 2020). Exogenous opioid exposure can regulate DOPR trafficking in a similar way to the induction of pain. For example, morphine exposure increases DOPR expression at the cell surface of DRG or cortical neurons (Cahill et al., 2001; Morinville et al., 2004b; Gendron et al., 2006). DOPR may also play an important role in the development of analgesic tolerance to exogenous opioids because genetic deletion of DOPR or DOPR antagonists can prevent the development of analgesic tolerance to morphine (Zhu et al., 1999; Abul-Husn et al., 2007; Beaudry et al., 2015). However, the role of DOPR in regulating opioid reward is less clear. DOPR knock-out or DOPR antagonists can facilitate morphine-induced locomotor sensitization (Chefer and Shippenberg, 2009; Billa et al., 2010), a measure of drug responsivity manifesting after repeated drug exposures. However, similar DOPR manipulations have been shown to reduce morphine CPP (Chefer and Shippenberg, 2009; le Merrer et al., 2009, 2011; Billa et al., 2010). These effects may not be attributed to reductions in opioid reinforcement, *per se*, as these manipulations fail to alter morphine self-administration (David et al., 2008; le Merrer et al., 2011). Instead, DOPR may play an important role in drug-cue associated learning.

DOPR signaling is necessary for cued value-based decisions making, particularly within the NAc shell (Laurent et al., 2014, 2012). This effect is driven by distinct anatomical regulation of DA transmission in the NAc by DOPR (Saigusa et al., 2017), such that DOPR agonists in the NAc core increase extracellular DA, while decreasing DA release in the NAc shell (Hirose et al., 2005; Hipólito et al., 2008; Saigusa et al., 2017). Adding another layer of complexity to DOPR-mediated effects on DA release, is that distinct DOPR subtypes (DOPR-1 and DOPR-2) can differentially regulate DA release through their interactions with MOPR. While stimulation of either subtype can have an inhibitory influence over MOPR-mediated slow increases DA release, the precise mechanisms underlying these effects are less clear. For example, stimulation of DOPR-1, not DOPR-2, can activate MOPR causing rapid increases in extracellular DA. However, DOPR agonists can also facilitate DA release independent of MOPR or DOPR, possibly by regulating sodium channel activity (Murakawa et al., 2004; Hirose et al., 2005; Saigusa et al., 2017). In contrast, DOPR-2, not DOPR-1, may play an important role in the development of analgesic tolerance (Beaudry et al., 2015). Future research delineating the precise role of DOPR in mesolimbic circuits will be crucial to exploit on the therapeutic potential of targeting the DOPR system for pain and opioid abuse. Interestingly, gene polymorphisms to the DOPR encoding gene have been linked with increased risk for drug dependence, further strengthening the need for untangling the DOPR system from the behavioral to the genetic level (Zhang et al., 2008; Crist et al., 2013). Moreover, because DOPR agonists have lower abuse liability than MOPR agonists (Stevenson et al., 2005), the DOPR system may represent a useful target for managing pain states during long-term opioid therapy. While the analgesic properties and anxiolytic effects of DOPR agonists are desirable for improving mood states of chronic pain patients, it should be noted that activation of DOPRs can lead to convulsions which may limit their clinical utility (Pradhan et al., 2011). As such, advancing clinical use of DOPR-based ligands will likely be dependent on the development of biased-ligands or dimer-specific drugs capable of DOPR heteromized with other GPCRs (Chu Sin Chung and Kieffer, 2013). Nevertheless, the DOPR system represents a promising target for the development of chronic pain therapies with improved analgesia and minimal unwanted side-effects attributed to traditional opioid medications.

### Nociceptin/orphanin FQ opioid receptor

The role of the NOPR system in pain is complex (Toll et al., 2016). In animal models of inflammatory pain, neuropathic pain, and fibromyalgia, NOPR expression and respective endogenous peptide, nociceptin/orphanin FQ (N/OFQ), are upregulated in DRG neurons, spinal tissue, and supraspinal sites (Andoh et al., 1997; Briscini et al., 2002; Dagnino et al., 2019). The ability of NOPR to regulate nociception is related to crosstalk between the NOPR system and stress systems and anatomical distinctions in NOPR function in spinal versus supraspinal sites. Early studies found that intracerebroventricular administration of N/OFQ reduced hot plate and tail flick latencies, suggesting a pro-nociceptive role of supraspinal NOPR activity (Meunier et al., 1995). However, subsequent studies determined that this pro-nociceptive effect was solely related to stress-induced analgesia (Mogil et al., 1996a,b; Morgan, 1997; Rizzi et al., 2001, 2007), a phenomenon triggering the release of endogenous opioids. The pro-nociceptive effects supraspinal N/OFQ are driven partially by antagonistic effects at MOPR, DOPR, and KOPR (Mogil et al., 1996a,b) as well as non-opioid components of stress-induced analgesia (Rizzi et al., 2001). On the contrary, intrathecal administration of N/OFQ produces anti-nociceptive effects and potentiates the effects of morphine (Xu et al., 1996; Yamamoto et al., 1997), indicating the role of NOPR signaling in pain is anatomically specific. Intrathecal administration of N/OFQ or NOPR agonists reduce pain sensitivity in animal models of neuropathic and inflammatory pain (Hao et al., 1998; Ko and Naughton, 2009; Tzschentke et al., 2017). Similar effects are observed with systemic NOPR agonists on mechanical allodynia in preclinical models of cancer-induced bone pain (Sliepen et al., 2021).

NOPR function also varies depending on the persistence of pain. Genetic deletion of NOPR does not alter acute pain sensitivity but exacerbates hyperalgesic responses in conditions of persistent inflammatory pain (Depner et al., 2003; Rizzi et al., 2011). However, significant differences in NOPR supraspinal distribution and localization is observed between species, particularly between preclinical animal models and non-human primates/humans (Florin et al., 2000; Berthele et al., 2003). As such, the effects of NOPR manipulations in preclinical models of pain may not directly translate to clinical populations (Spetea et al., 2022). Because cellular adaptations within the NOPR system and anatomical distribution of NOPR vary across species and different pain models, future research is required to uncover how recruiting/silencing NOPR signaling can efficiently treat pain symptoms in a more individualized setting.

When considering the clinical utility of targeting the NOPR system for treating opioid abuse in pain patients, it is important to highlight that NOPR activity is neither rewarding nor aversive (Devine et al., 1996). This significantly adds to the therapeutic potential of targeting the NOPR system since NOPR manipulations mitigate abuse potential while sparing negative side-effects. NOPR agonists reduce extracellular release of DA in the NAc (Murphy et al., 1996; Lutfy et al., 2001a), suggesting an inhibitory influence of NOPR activity over drug reward. Indeed, intracerebroventricular administration of N/OFQ or NOPR agonists block the acquisition of CPP for morphine, cocaine, alcohol, and methamphetamine (Ciccocioppo et al., 2000; Kotliñska et al., 2002, Kotlinska et al., 2003; Sakoori and Murphy, 2004; Zaveri et al., 2018). This evidence further solidifies the therapeutic potential of the NOPR system in mitigating opioid abuse and substance use disorders in general. Recent studies found that local administration of N/OFQ in the central amygdala attenuates escalation of oxycodone self-administration (Kallupi et al., 2020). These effects may be attributed to site-specific NOPR regulation as intracerebroventricular administration of N/OFQ fails to reduce heroin self-administration (Walker et al., 1998.). Further adding to this complexity is that the effects of NOPR manipulations have inconsistent effects on alcohol self-administration (Ciccocioppo et al., 1999, 2004; Kuzmin et al., 2004; Economidou et al., 2008). One possibility is that NOPR function may be important for drug-associated memory formation given that NOPR activity can negatively impact memory (Moulédous, 2019). In this regard, NOPR activity may impact the formation of drug-context association (conditioned place preference) rather than impact drug reinforcement and thus, instrumental drug-seeking behavior. This would align with findings demonstrating that NOPR agonists effectively block the acquisition of morphine CPP, but not its expression (Shoblock et al., 2005). The precise mechanisms underlying the effects of pain and opioid use on NOPR function remain unclear, but emerging evidence indicates that NOPR agonists, like cebranopadol, have high analgesic efficacy in chronic pain, delayed development of analgesic tolerance, and lower abuse potential (Linz et al., 2014; Tzschentke et al., 2019). Therefore, it will be important for ongoing research endeavors to fully characterize the role of NOPR in the context of pain and opioid misuse liability and determine whether this opioid system is a therapeutic target with clinical utility.

## Opioid system dysfunction by exogenous opioids

Chronic exogenous opioid use can lead to the development of tolerance, a progressive decrease in opioid efficacy which can be mitigated by increasing opioid doses (Lee et al., 2011). Pain patients on long-term opioid therapy typically require increasing doses of opioids to achieve the same level of analgesia (Williams et al., 2001; Zernig et al., 2007; Hayes et al., 2020). In addition to analgesia, tolerance to other opioid-induced effects, like euphoria, sedation, nausea, respiratory depression, and constipation, can also develop over time, albeit not at the same rate (Hayhurst and Durieux, 2016). For example, the development of analgesic and euphoric tolerance occurs on a faster time scale than tolerance to respiratory depression (Ling et al., 1989; Volkow et al., 2018), which contributes to the heightened risk of overdose for opioid users with escalating opioid doses (Kaplovitch et al., 2015; Hayes et al., 2019, 2020). Furthermore, the rate at which tolerance develops often depends on genetic variability and differential responses to different opioid ligands, duration of exposure, and route of administration (Dumas and Pollack, 2008; Ballantyne and Koob, 2021).

### Tolerance

The development of tolerance stems from desensitization of the opioid system and inflammatory immune responses within peripheral and central nervous systems (Zhu et al., 1999; Dumas and Pollack, 2008; Koch and Höllt, 2008; Matsui et al., 2014; Corder et al., 2017; Lueptow et al., 2018; Eidson and Murphy, 2019). Following activation, opioid receptors can be phosphorylated by GPCR kinases, which triggers G-protein uncoupling and binding of β-arrestins (Dumas and Pollack, 2008; Zhou et al., 2021). β-arrestin pathway signaling causes desensitized receptors to remain inactive at the plasma membrane, facilitates their endocytosis and subsequent degradation or recycling. As such, these cellular mechanisms represent a critical component in facilitating the development of tolerance at multiple levels (Hutchings et al., 1997; Bohn et al., 2000; Koch and Höllt, 2008; Zhou et al., 2021). Biased agonists, that preferentially activate G-protein signaling cascades with minimal β-arrestin pathway activity, have received great interest as therapeutic alternatives with the thought that such ligands may minimize the development of tolerance and other unwanted side-effects (Ballantyne and Chavkin, 2020). In mice with genetic deletion of the β-arrestin2 isoform, acute morphine prolongs analgesia while reducing the unwanted side-effects of respiratory depression and constipation, while chronic morphine treatment reduces MOPR desensitization and the development of tolerance (Bohn et al., 1999, 2000; Raehal et al., 2011). These findings led to the development of functionally selective MOPR agonists, like oliceridine, which exhibit preference for G protein-biased signaling and produce less respiratory depression in preclinical models compared to non-selective agonists (DeWire et al., 2013). However, subsequent studies found that opioid-induced respiratory depression and constipation may occur independent of β-arrestins (Kliewer et al., 2020) and G-protein selectivity may worsen some side effects (Kliewer et al., 2019). Although negative side-effects remained during clinical trials (Hertz, 2018), the risk for respiratory depression with oliceridine was lower than morphine (Dahan et al., 2020). Similar findings were found for another biased-MOPR agonist, PZM21 (Graeme Henderson et al., 2018), further highlighting the need to better understand biased opioid ligand signaling mechanisms and their role tolerance.

NOPR signaling appears to play facilitative role in the development of tolerance in the context of pain. As previously mentioned, NOPR expression increases after the induction of pain in spinal and supraspinal sites (Andoh et al., 1997; Briscini et al., 2002; Dagnino et al., 2019) which, under conditions of chronic pain, can suppress hyperalgesic responses (Depner et al., 2003; Rizzi et al., 2011). Based on this, it is somewhat surprising that N/OFQ potentiates the development of opioid tolerance. Genetic ablation of the endogenous peptide, nociceptin, N/OFQ, its receptor (NOPR), or blocking NOPR signaling using an exogenous antagonist, prevents and reverses the development of morphine tolerance (Ueda et al., 1997; Lutfy et al., 2001b; Chung et al., 2006; Scoto et al., 2010). These likely are attributed to the antagonistic properties of N/OFQ at other opioid receptors (Mogil et al., 1996b). NOPR can also undergo desensitization after chronic or acute stimulation (Donica et al., 2013). While these findings suggest that pain-induced upregulation of the NOPR system underlies attenuated analgesic responses to exogenous opioids, they also suggest that targeting the NOPR system may be a useful target to treat vulnerabilities in opioid tolerance, escalation, and abuse in pain patients.

The development of tolerance can also develop in response to the recruitment of neuroinflammatory mediators. Long-term opioid use triggers neuroinflammatory responses in the CNS to increase neuronal excitability which can contribute to tolerance (Eidson and Murphy, 2019; Zhang et al., 2020; Zhou et al., 2021). In particular, the ventrolateral PAG (vlPAG) is a critical hub in which descending control over nociceptive signaling is negatively affected by chronic opioid use. Chronic intra-vlPAG opioid agonist administration is sufficient to produce tolerance to systemically administered opioids. Similarly, blocking vlPAG opioid receptor-mediated signaling can prevent the development of tolerance to chronic systemic administration of exogenous opioids (Lane et al., 2004; Morgan et al., 2006; Meyer et al., 2007; Loyd et al., 2008; Macey et al., 2009; Bobeck et al., 2012; Eidson and Murphy, 2019). Opioid-induced activation of toll-like receptor 4 (TLR4) in astrocytes and microglia within the spinal cord or PAG triggers inflammatory responses through activation of nuclear factor kappa B (NFκB) and the release of pro-inflammatory cytokines such as tumor necrosis factor α (TNFα) and interleukins, IL-1β and IL-6 (Raghavendra et al., 2002; Eidson et al., 2016; Liang et al., 2016; Eidson and Murphy, 2019; Wang et al., 2020). This release in cytokines leads to down-regulation of GABA receptors resulting in increased function of glutamate receptor systems. Consequently, hyper-excitability in nociceptive pathways acts to oppose the analgesic actions of opioids, resulting in tolerance (DeLeo et al., 2004; Eidson and Murphy, 2019; Zhou et al., 2021). Based on the role of cytokines in opioid tolerance, significant efforts have been directed toward the development of treatments that may inhibit opioid-induced cytokine production (Namba et al., 2021). For example, manipulations inhibiting TNF signaling through TLR4 can prevent morphine tolerance and associated hyperexcitability (Shen et al., 2011; Eidson et al., 2016; Wang et al., 2020). As such, modulation of TNF signaling represents a promising adjunctive therapy to curb the development of opioid tolerance. Taken together, opioid tolerance manifests through adaptations to endogenous opioid and inflammatory systems, but a better understanding of the relationship between these systems will facilitate our ability to identify novel therapeutic targets to overcome the development of opioid tolerance.

### Opioid-induced hyperalgesia

In contrast to the development of tolerance, chronic opioid use can also result in opioid-induced hyperalgesia (OIH), a paradoxical increase in pain sensitivity either at the initial source of pain or elsewhere (Chu et al., 2008; Hay et al., 2009; Roeckel et al., 2016). While the phenomenon of tolerance represents a reduction in drug potency and creates a rightward shift in analgesic opioid dose response curves, OIH increases pain sensitivity modeled by a significant downward shift in analgesic dose response (Chu et al., 2008). It is, thus, distinct from tolerance in that escalating opioid doses may exacerbate the development of OIH in the long-term. However, both tolerance and OIH are associated with hyperexcitability in glutamatergic systems and up-regulation of pro-inflammatory molecules at spinal synapses and supraspinal regions, like the RVM (Bederson et al., 1990; Kaplan and Fields, 1991; Kovelowski et al., 2000; Vanderah et al., 2001). OIH is a pro-nociceptive process that can be observed independently of tolerance through acute exposure to ultra-low opioid doses. However, the development of OIH is more often observed after the development of tolerance, following chronic exposure to higher, analgesic doses (Drdla et al., 2009; Silverman, 2009; Lee et al., 2011; Hayhurst and Durieux, 2016; Roeckel et al., 2016).

Opioid-induced hyperalgesia is driven by cellular adaptations in pronociceptive signaling pathways, particularly within glutamatergic systems (Lee et al., 2011). Opioid agonists increase cellular excitability underlying OIH by inhibiting glutamate transporter systems (Mao et al., 2002). The resulting abundance in synaptic glutamate can lead to NMDA receptor-dependent long-term potentiation (LTP) at primary afferents and second-order spinal neurons resulting in sensitization of pain signaling pathways (Drdla et al., 2009; Silverman, 2009; Heinl et al., 2011; Drdla-Schutting et al., 2012; Roeckel et al., 2016; Corder et al., 2018). Adding to this, previous reports from our lab found that OIH is driven by insertion of GluA4-containing AMPA receptors in the dorsal horn of the spinal cord (Cabañero et al., 2013). Similar to the development of tolerance, OIH is also associated with opioid-dependent production and release of nociceptive signaling molecules from microglia and astrocytes such as pro-inflammatory cytokines, chemokines, ATP, nitric oxide, and others detailed elsewhere (Chu et al., 2008; Lee et al., 2011; Roeckel et al., 2016). Consequent release of the neuropeptide, cholecystokinin (CCK) in the RVM has been shown to have ‘anti-opioid’ actions that facilitate pronociceptive pathways contributing to OIH (Kaplan and Fields, 1991; Kovelowski et al., 2000; Friedrich and Gebhart, 2003; Heinricher and Neubert, 2004). The NMDA receptor-dependent hyperexcitability associated with OIH has been targeted in efforts to mitigate the impact of opioids on central sensitization. For example, low-dose ketamine (non-selective NMDA receptor antagonist) administration in conjunction with opioid analgesics can prevent the development of OIH in animal models and clinical patients with postoperative pain (Célèrier et al., 2000; Maher et al., 2017). Similarly, methadone, a potent MOPR agonist and weak NMDA receptor antagonist, has been examined as a substitute for opioid therapies and can effectively reduce opioid-induced OIH (Sjøgren et al., 1994; Shimoyama et al., 1997; Davis and Inturrisi, 1999; Axelrod and Reville, 2007). While the efficacy of methadone maintenance treatment (MMT) is less reliable in patients with opioid dependence or a prior history of abuse, MMT reduce instances of heroin use, drug craving, and criminal activity (Dole and Nyswander, 1965, 1976; Shi et al., 2008; Mattick et al., 2009; Ward et al., 2009; Lee et al., 2011). Despite this, moral reservations among some groups precipitated shifts in the treatment goals initially outlined for long-term MMT advising sufficient dosing and instead, goals were centered around achieving abstinence and using less-than-effective doses, which compromised treatment outcomes and funding for MMT research (Dole and Nyswander, 1976; Ward et al., 2009). As such, OIH remains a barrier to effective treatment with opioids. Further research delineating the mechanisms mediating the physiological and behavioral effects of opioids and whether pain affects these properties will help facilitate the development of novel and safer pharmacotherapies to improve patient care and well-being.

### Pain, opioids, and reward

The mesolimbic pathway integrates both aversive and rewarding properties of external stimuli (Bromberg-Martin et al., 2010). Activation of the mesolimbic pathway by rewarding stimuli results in phasic DA release from the VTA into the NAc to reinforce goal-directed behaviors (Fibiger et al., 1987; Berridge and Robinson, 1998; Becerra and Borsook, 2008; Pignatelli and Bonci, 2015). As described previously, opioids reliably activate mesolimbic DA pathway and thus promote motivational salience (Matsui et al., 2014; Galaj et al., 2020; Doyle and Mazei-Robison, 2021). In conditions of pain, the ability of opioids to trigger comparable responses is significantly reduced. Furthermore, the motivational salience of opioid reward may be driven by hedonic pleasure (positive reinforcement) or pain relief (negative reinforcement) (Koob, 2020). Similar to exogenous opioids in pain-naïve conditions, relief from pain itself can elicit increases in DA release and reinforce motivated behaviors (Martin et al., 2006; Leknes et al., 2011; Navratilova et al., 2015; Eikemo et al., 2021). As such, the presence of pain may perpetuate maladaptive patterns of opioid use.

Pain disrupts mesolimbic DA function contributing to maladaptive effects on reward processing. Deficits in DA signaling, or administration of DA receptor antagonists reduce approach behaviors and hedonic responses to rewarding stimuli (Frank et al., 2016; Nguyen et al., 2019). In rodent models of inflammatory and nerve injury pain, motivated behaviors for natural and drug rewards, such as opioids, are significantly impaired (Schwartz et al., 2014; Hipólito et al., 2015; Taylor et al., 2015; Massaly et al., 2019). This pain-induced decrease in motivation is strongly correlated with blunted DA signaling in the mesolimbic pathway (Cahill et al., 2013; Schwartz et al., 2014; Hipólito et al., 2015). These findings parallel clinical studies in which pain-induced negative emotional states positively correlates with reductions in DA neurotransmission and maladaptive changes in NAc function (Lee and Tracey, 2010; Jarcho et al., 2012; Martikainen et al., 2015; Makary et al., 2020). Importantly, pain-related alterations in DA signaling are also associated with deficits in emotional and sensory processing. For example, deficits in DA receptor binding potential in the NAc are observed in patients with lower back pain, which can predict the severity of negative affect and pain (Baliki et al., 2010; Martikainen et al., 2015). In line with this, DA transporter activity, a mechanism important for clearing DA from the synaptic cleft, is increased in the NAc of animal models of chronic neuropathic or inflammatory pain (Ren et al., 2015, 2021; Selley et al., 2020). Moreover, morphine-induced DA release in the NAc is suppressed by sciatic nerve ligation (Niikura et al., 2008). These changes in mesolimbic DA function strongly impact reward thresholds which may contribute to pain-related occurrences of negative affect and enhanced vulnerability for opioid abuse (Massaly et al., 2019, 2021). Supporting this, pain patients are more likely to initiate and continue opioid treatment if they have a cooccurring mood disorder (Halbert et al., 2016).

Opioid abuse susceptibility in pain states is likely exacerbated by a rightward shift in opioid reinforcement thresholds due to pain-related deficits in mesolimbic pathway function. In lower back pain patients, the propensity for risky monetary behavior is associated with altered connectivity of the NAc (Berger et al., 2014). The severity of pain is also associated with increased impulsivity in humans and rodent models (Wakaizumi et al., 2019; Cunha et al., 2020). These would suggest that pain patients are predisposed to developing problematic opioid use. Although it is recognized that chronic pain patients receiving prescription opioids are at high risk for opioid dependence (Ballantyne, 2015), the prevalence of maladaptive opioid use in pain patients has been difficult to determine based on confounding outcome measurements (i.e., mortality) and imprecise or poorly defined terminology (i.e., “abuse,” “misuse,” “addiction”) (Vowles et al., 2015). Opioid “misuse,” or use contrary to the prescribed pattern, occurs in up to 29% of pain patients receiving opioid medications while “addiction,” or continued use despite negative consequences, can occur in up to 12% (Vowles et al., 2015). Opioid “abuse,” or aberrant drug taking behavior often predictive of maladaptive opioid use has been reported in 46–81% of pain populations (Butler et al., 2004; Wilsey et al., 2008; Vowles et al., 2015). However, there remains a general consensus that high-quality research on this relationship is lacking (Ballantyne, 2015; Voon et al., 2017; Nadeau et al., 2021). Nevertheless, qualitative evidence from clinical literature indicates that negative outcomes associated with opioid use can be instigated by pain severity (Grol-Prokopczyk, 2017; Zajacova et al., 2021), duration of opioid use (Chung et al., 2019; Jantarada et al., 2021), escalating opioid doses (Zernig et al., 2007; Kaplovitch et al., 2015), comorbid anxiety and depression (Peciña et al., 2018; Emery and Akil, 2020; Rogers et al., 2020), discontinuation of opioid medications (Mark and Parish, 2019; Stein et al., 2021), and inherent risk factors like sex (Manubay et al., 2015; McHugh, 2020) or genetics (Kendler et al., 2003; Agarwal et al., 2017). Evidence from patients with pain and long-term opioid use have been critical in identifying potential risk factors for maladaptive opioid use but have yielded minimal impacts on either public health concern.

Determining the level of synergy between pain, long-term opioid use, and opioid misuse can be difficult for many reasons, but preclinical pain models of opioid self-administration provide a translational means to better understand how pain may provoke motivational shifts to alter opioid misuse liability. Although pain-induced dysfunction of mesolimbic reward pathways produces clear deficits in motivation for natural rewards (Massaly et al., 2019, 2021; Reiner et al., 2019), the effects of pain on opioid motivation are more complex. Evidence from self-administration studies suggest that the ability of pain to effect opioid self-administration is related to the chronicity of pain, selected opioid/dose, and the duration of daily opioid exposure. For example, chronic arthritic pain has biphasic effects on rates of oral fentanyl self-administration, that interestingly, follow the time-course of pain progression (Colpaert et al., 2001, 1982). Specifically, one week after the onset of pain there are no effects on fentanyl consumption but, during successive weeks, fentanyl intake dramatically increases—peaking at 2–3 weeks – and declines to baseline levels several weeks later. Importantly, the time course of fentanyl consumption rates parallels the time course of progressive pain sensitivity (Colpaert et al., 1982, 2001). Similarly, spinal cord injury has time-dependent effects on long-access morphine self-administration in rats. In this regard, pain reduces morphine intake 24 h after the induction of pain, then peaks at 14–21 days before normalizing 35–42 days later (Woller et al., 2014). These findings indicate that the persistence of pain is an important driver of opioid consumption. Adding further support to this, acute pain manipulations with capsaicin or lactic acid do not alter rates of fentanyl or heroin self-administration, but persistent inflammatory pain-induced reductions in fentanyl vs. food choice procedures match controls by one week after the induction of pain (Reiner et al., 2021). Notably, a small study found that arthritic pain reduced self-administration of relatively high doses of morphine with 24-h access for weeks following pain onset (Lyness et al., 1989) while another found that multiple forms of chronic pain attenuated oral fentanyl self-administration and discrimination in mice (Wade et al., 2013). These findings allude to the notion that pain may produce a shift opioid dose-response. Consistent with this, our lab found that inflammatory pain reduces heroin intake at low doses, but increases intake when doses are high (Hipólito et al., 2015). Our findings suggest that these effects are driven by deficits in VTA DA cell excitability (Hipólito et al., 2015) and this is exemplified by evidence showing that pain reduces the ability of low-dose opioids to facilitate VTA intracranial self-stimulation (Ewan and Martin, 2011). Spinal nerve ligation also produces a rightward shift in dose-response for multiple opioids, but the time-dependency of these effects has not been examined (Martin et al., 2007). Taken together, evidence from preclinical pain models of opioid abuse suggest that chronic pain can increase motivation for high opioid doses in a time-dependent manner that parallels the progression of pain. It will be important for future studies to evaluate whether the time- and dose-dependent effects of pain on opioid consumption are related to time-dependent disruptions in mesolimbic pathway function.

## Conclusions

Pain conditions, chronic opioid use, and withdrawal from chronic opioid use disrupt the endogenous opioid system function at spinal and supraspinal levels to negatively impact pain thresholds, opioid sensitivity, mood, and reward sensitivity. These physiological and behavioral alterations, particularly among opioid systems and mesolimbic reward pathways, may contribute to persistent use of opioid medications in an attempt to alleviate adverse physical and emotional states, thereby creating a susceptibility for opioid misuse. In addition, other mediating factors outside the scope of this review contribute to individual variabilities in pain perception and opioid sensitivity like sex differences (Huhn et al., 2018; Pisanu et al., 2019), genetic (Tremblay and Hamet, 2010; Mogil, 2012), and epigenetic mechanisms (Liang et al., 2015; Browne et al., 2019) and likely influence proclivity for opioid abuse in the context of pain. Neuroadaptive processes produced by pain conditions and long-term opioid use have compounding effects on negative outcomes, like the development of tolerance or opioid-induced hyperalgesia. An understanding of the synergy between these processes remains incomplete, but the ability to curb the opioid crisis and the prevalence of pain relies heavily on the ability to identify safer pharmacotherapeutic alternatives derived from a better comprehension of pain- and opioid-induced adaptations to opioid systems and functional neurocircuitry.

## Author contributions

All authors contributed to the format and writing of the review and approved the final submitted version.

## References

[B1] Abul-Husn N. S. Sutak M. Milne B. Jhamandas K. (2007). Augmentation of spinal morphine analgesia and inhibition of tolerance by low doses of mu- and delta-opioid receptor antagonists. *Br. J. Pharmacol.* 151 877–887. 10.1038/SJ.BJP.0707277 17502848 PMC2014123

[B2] Agarwal D. Udoji M. A. Trescot A. (2017). Genetic testing for opioid pain management: a primer. *Pain Ther.* 6 93–105. 10.1007/S40122-017-0069-2 28409480 PMC5447546

[B3] Ahmad F. Rossen L. Sutton P. (2022). *Provisional Drug Overdose Death Counts.* Hyattsville, MD: National Center for Health Statistics.

[B4] Ahmadi J. Jahromi M. S. Ehsaei Z. (2018a). The effectiveness of different singly administered high doses of buprenorphine in reducing suicidal ideation in acutely depressed people with co-morbid opiate dependence: a randomized, double-blind, clinical trial. *Trials* 19 1–8. 10.1186/s13063-018-2843-9 30157924 PMC6114789

[B5] Ahmadi J. Jahromi M. S. Ghahremani D. London E. D. (2018b). Single high-dose buprenorphine for opioid craving during withdrawal. *Trials* 19 1–7. 10.1186/S13063-018-3055-Z/TABLES/4 30526648 PMC6288888

[B6] Al-Hasani R. Bruchas M. R. (2011). Molecular mechanisms of opioid receptor-dependent signaling and behavior. *Anesthesiology* 115 1363–1381. 10.1097/ALN.0B013E318238BBA6 22020140 PMC3698859

[B7] Al-Hasani R. McCall J. G. Shin G. Gomez A. M. Schmitz G. P. Bernardi J. M. (2015). Distinct subpopulations of nucleus accumbens dynorphin neurons drive aversion and reward. *Neuron* 87 1063–1077. 26335648 10.1016/j.neuron.2015.08.019 PMC4625385

[B8] Andoh T. Itoh M. Kuraishi Y. (1997). Nociceptin gene expression in rat dorsal root ganglia induced by peripheral inflammation. *Neuroreport* 8 2793–2796. 10.1097/00001756-199708180-00028 9295119

[B9] Axelrod D. J. Reville B. (2007). Using methadone to treat opioid-induced hyperalgesia and refractory pain. *J. Opioid Manag.* 3 113–114. 10.5055/JOM.2007.0048 17520991

[B10] Baliki M. N. Geha P. Y. Fields H. L. Apkarian A. V. (2010). Predicting value of pain and analgesia: nucleus accumbens response to noxious stimuli changes in the presence of chronic pain. *Neuron* 66 149–160. 10.1016/J.NEURON.2010.03.002 20399736 PMC2873199

[B11] Ballantyne J. C. (2015). Assessing the prevalence of opioid misuse, abuse, and addiction in chronic pain. *Pain* 156 567–568. 10.1097/J.PAIN.0000000000000105 25630030

[B12] Ballantyne J. C. Chavkin C. (2020). Will biased ligands be the opioids of the future? *Pain* 161 1945–1948. 10.1097/J.PAIN.0000000000001913 32379220

[B13] Ballantyne J. C. Koob G. F. (2021). Allostasis theory in opioid tolerance. *Pain* 162 2315–2319. 10.1097/J.PAIN.0000000000002280 33769368

[B14] Ballester J. Baker A. K. Martikainen I. K. Koppelmans V. Zubieta J. K. Love T. M. (2022). Risk for opioid misuse in chronic pain patients is associated with endogenous opioid system dysregulation. *Transl. Psychiatry* 12 1–7. 10.1038/s41398-021-01775-z 35022382 PMC8755811

[B15] Bannister K. Dickenson A. H. (2020). Central nervous system targets: supraspinal mechanisms of analgesia. *Neurotherapeutics* 17 839–845. 10.1007/S13311-020-00887-6 32700132 PMC7609795

[B16] Barrio P. Ortega L. Guardia J. Roncero C. Yuguero L. Gual A. (2018). Who receives nalmefene and how does it work in the real world? A single-arm, phase iv study of nalmefene in alcohol dependent outpatients: baseline and 1-month results. *Clin. Drug Investig.* 38 147–155. 10.1007/S40261-017-0590-4 29080208

[B17] Basbaum A. I. Fields H. L. (1984). Endogenous pain control systems: brainstem spinal pathways and endorphin circuitry. *Annu. Rev. Neurosci.* 7 309–338. 10.1146/ANNUREV.NE.07.030184.001521 6143527

[B18] Beaudry H. Gendron L. Morón J. A. (2015). Implication of delta opioid receptor subtype 2 but not delta opioid receptor subtype 1 in the development of morphine analgesic tolerance in a rat model of chronic inflammatory pain. *Eur. J. Neurosci.* 41 899–905. 10.1111/ejn.12829 25639561 PMC4390435

[B19] Becerra L. Borsook D. (2008). Signal valence in the nucleus accumbens to pain onset and offset. *Eur. J. Pain* 12 866–869. 10.1016/J.EJPAIN.2007.12.007 18226937 PMC2581873

[B20] Beckett A. H. Casy A. F. (1954). Synthetic analgesics: stereochemical considerations. *J. Pharm. Pharmacol.* 6 986–1001. 10.1111/J.2042-7158.1954.TB11033.X 13212680

[B21] Bederson J. B. Fields H. L. Barbaro N. M. (1990). Hyperalgesia during naloxone-precipitated withdrawal from morphine is associated with increased on-cell activity in the rostral ventromedial medulla. *Somatosens. Mot. Res.* 7 185–203. 10.3109/08990229009144706 2378192

[B22] Bencherif B. Fuchs P. N. Sheth R. Dannals R. F. Campbell J. N. Frost J. J. (2002). Pain activation of human supraspinal opioid pathways as demonstrated by [11C]-carfentanil and positron emission tomography (PET). *Pain* 99 589–598. 10.1016/S0304-3959(02)00266-X 12406535

[B23] Berger S. E. Baria A. T. Baliki M. N. Mansour A. Herrmann K. M. Torbey S. (2014). Risky monetary behavior in chronic back pain is associated with altered modular connectivity of the nucleus accumbens. *BMC Res. Notes* 7:739. 10.1186/1756-0500-7-739 25331931 PMC4210520

[B24] Berridge K. C. Robinson T. E. (1998). What is the role of dopamine in reward: hedonic impact, reward learning, or incentive salience? *Brain Res. Rev.* 28 309–369. 10.1016/S0165-0173(98)00019-8 9858756

[B25] Berthele A. Platzer S. Dworzak D. Schadrack J. Mahal B. Büttner A. (2003). [3H]-nociceptin ligand-binding and nociceptin opioid receptor mrna expression in the human brain. *Neuroscience* 121 629–640. 10.1016/S0306-4522(03)00484-6 14568023

[B26] Bilkei-Gorzo A. Michel K. Noble F. Roques B. P. Zimmer A. (2007). Preproenkephalin knockout mice show no depression-related phenotype. *Neuropsychopharmacology* 32 2330–2337. 10.1038/SJ.NPP.1301370 17375141

[B27] Billa S. K. Xia Y. Morón J. A. (2010). Disruption of morphine-conditioned place preference by a delta2-opioid receptor antagonist: study of mu-opioid and delta-opioid receptor expression at the synapse. *Eur. J. Neurosci.* 32 625–631. 10.1111/J.1460-9568.2010.07314.X 20626460 PMC3596814

[B28] Bloms-Funke P. Gillen C. Schuettler A. J. Wnendt S. (2000). Agonistic effects of the opioid buprenorphine on the nociceptin/OFQ receptor. *Peptides* 21 1141–1146. 10.1016/S0196-9781(00)00252-7 10998549

[B29] Blondell R. D. Ashrafioun L. Dambra C. M. Foschio E. M. Zielinski A. L. Salcedo D. M. (2010). A clinical trial comparing tapering doses of buprenorphine with steady doses for chronic pain and coexistent opioid addiction. *J. Addict. Med.* 4 140–146. 10.1097/ADM.0b013e3181ba895d 20959867 PMC2931595

[B30] Bobeck E. N. Haseman R. A. Hong D. Ingram S. L. Morgan M. M. (2012). Differential development of antinociceptive tolerance to morphine and fentanyl is not linked to efficacy in the ventrolateral periaqueductal gray of the rat. *J. Pain* 13 799–807. 10.1016/J.JPAIN.2012.05.005 22766006 PMC3958948

[B31] Bodnar R. J. (2021). Endogenous opiates and behavior: 2019. *Peptides* 141:170547. 10.1016/J.PEPTIDES.2021.170547 33831447

[B32] Bohn L. M. Gainetdinov R. R. Lin F. T. Lefkowitz R. J. Caron M. G. (2000). μ-Opioid receptor desensitization by β-arrestin-2 determines morphine tolerance but not dependence. *Nature* 408 720–723. 10.1038/35047086 11130073

[B33] Bohn L. M. Lefkowitz R. J. Gainetdinov R. R. Peppel K. Caron M. G. Lin F. T. (1999). Enhanced morphine analgesia in mice lacking β-arrestin 2. *Science* 286 2495–2498. 10.1126/science.286.5449.2495 10617462

[B34] Borsook D. Linnman C. Faria V. Strassman A. M. Becerra L. Elman I. (2016). Reward deficiency and anti-reward in pain chronification. *Neurosci. Biobehav. Rev.* 68 282–297. 10.1016/J.NEUBIOREV.2016.05.033 27246519

[B35] Bourinet E. Soong T. W. Stea A. Snutch T. P. (1996). Determinants of the G protein-dependent opioid modulation of neuronal calcium channels. *Proc. Natl. Acad. Sci. U.S.A.* 93 1486–1491. 10.1073/pnas.93.4.1486 8643659 PMC39966

[B36] Briscini L. Corradini L. Ongini E. Bertorelli R. (2002). Up-regulation of ORL-1 receptors in spinal tissue of allodynic rats after sciatic nerve injury. *Eur. J. Pharmacol.* 447 59–65. 10.1016/s0014-2999(02)01833-2 12106803

[B37] Bromberg-Martin E. S. Matsumoto M. Hikosaka O. (2010). Dopamine in motivational control: rewarding, aversive, and alerting. *Neuron* 68 815–834. 10.1016/J.NEURON.2010.11.022 21144997 PMC3032992

[B38] Bronstein D. M. Przewlocki R. Akil H. (1990). Effects of morphine treatment on pro-opiomelanocortin systems in rat brain. *Brain Res.* 519 102–111. 10.1016/0006-8993(90)90066-K 2144463

[B39] Browne C. J. Godino A. Salery M. Nestler E. J. (2019). Epigenetic mechanisms of opioid addiction. *Biol. Psychiatry* 87 22–33. 10.1016/j.biopsych.2019.06.027 31477236 PMC6898774

[B40] Brownstein M. J. (1993). Review A brief history of opiates, opioid peptides, and opioid receptors. *Proc. Natl. Acad. Sci. U.S.A.* 90 5391–5393. 10.1073/pnas.90.12.5391 8390660 PMC46725

[B41] Bruchas M. R. Land B. B. Chavkin C. (2009). The dynorphin / kappa opioid system as a modulator of stress-induced and pro-addictive behaviors. *Brain Res.* 1314 44–55. 10.1016/j.brainres.2009.08.062 19716811 PMC2819621

[B42] Bruehl S. Burns J. W. Chung O. Y. Chont M. (2012). What do plasma beta-endorphin levels reveal about endogenous opioid analgesic function? *Eur. J. Pain* 16 370–380. 10.1002/j.1532-2149.2011.00021.x 22337161 PMC3786673

[B43] Bruehl S. Burns J. W. Gupta R. Buvanendran A. Chont M. Kinner E. (2013). Endogenous opioid function mediates the association between laboratory-evoked pain sensitivity and morphine analgesic responses. *Pain* 154 1856–1864. 10.1016/J.PAIN.2013.06.002 23748117 PMC4069065

[B44] Bruehl S. Burns J. W. Gupta R. Buvanendran A. Chont M. Orlowska D. (2017). Do Resting Plasma β-endorphin levels predict responses to opioid analgesics? *Clin. J. Pain* 33 12–20. 10.1097/AJP.0000000000000389 27183154 PMC5107361

[B45] Bruehl S. Burns J. W. Gupta R. Buvanendran A. Chont M. Schuster E. (2014). Endogenous opioid inhibition of chronic low back pain influences degree of back pain relief following morphine administration. *Reg. Anesth. Pain Med.* 39:120. 10.1097/AAP.0000000000000058 24553304 PMC3933525

[B46] Bunzow J. R. Saez C. Mortrud M. Bouvier C. Williams J. T. Low M. (1994). Molecular cloning and tissue distribution of a putative member of the rat opioid receptor gene family that is not a μ, δ or κ opioid receptor type. *FEBS Lett.* 347 284–288. 10.1016/0014-5793(94)00561-3 8034019

[B47] Burford N. T. Traynor J. R. Alt A. (2015). Positive allosteric modulators of the μ-opioid receptor: a novel approach for future pain medications. *Br. J. Pharmacol.* 172 277–286. 10.1111/BPH.12599 24460691 PMC4292945

[B48] Butler S. F. Budman S. H. Fernandez K. Jamison R. N. (2004). Validation of a screener and opioid assessment measure for patients with chronic pain. *Pain* 112 65–75. 10.1016/J.PAIN.2004.07.026 15494186

[B49] Cabañero D. Baker A. Zhou S. Hargett G. L. Irie T. Xia Y. (2013). Pain after discontinuation of morphine treatment is associated with synaptic increase of GluA4-containing AMPAR in the dorsal horn of the spinal cord. *Neuropsychopharmacology* 38 1472–1484. 10.1038/npp.2013.46 23403695 PMC3682142

[B50] Cahill C. M. Holdridge S. V. Liu S. Xue L. Magnussen C. Ong E. (2022a). Delta opioid receptor activation modulates affective pain and modality-specific pain hypersensitivity associated with chronic neuropathic pain. *J. Neurosci. Res.* 100 129–148. 10.1002/JNR.24680 32623788 PMC8218601

[B51] Cahill C. M. Lueptow L. Kim H. Shusharla R. Bishop A. Evans C. J. (2022b). Kappa opioid signaling at the crossroads of chronic pain and opioid addiction. *Handb. Exp. Pharmacol.* 271 315–350. 10.1007/164_2021_434 33547588 PMC9436635

[B52] Cahill C. M. Morinville A. Hoffert C. O’Donnell D. Beaudet A. (2003). Up-regulation and trafficking of δ opioid receptor in a model of chronic inflammation: implications for pain control. *Pain* 101 199–208. 10.1016/S0304-3959(02)00333-0 12507715

[B53] Cahill C. M. Morinville A. Lee M. C. Vincent J. P. Collier B. Beaudet A. (2001). Prolonged morphine treatment targets δ opioid receptors to neuronal plasma membranes and enhances δ-mediated antinociception. *J. Neurosci.* 21 7598–7607. 10.1523/JNEUROSCI.21-19-07598.2001 11567050 PMC6762923

[B54] Cahill C. M. Xue L. Grenier P. Magnussen C. Lecour S. Olmstead M. C. (2013). Changes in morphine reward in a model of neuropathic pain. *Behav. Pharmacol.* 24 207–213. 10.1097/FBP.0B013E3283618AC8 23591124

[B55] Carlezon W. A. Krystal A. D. (2016). Kappa-opioid antagonists for psychiatric disorders: from bench to clinical trials. *Depress Anxiety* 33 895–906. 10.1002/DA.22500 27699938 PMC5288841

[B56] CDC WONDER (2018). *CDC/NCHS, National Vital Statistics System, Mortality.* Atlanta, GA: CDC.

[B57] Célèrier E. Rivat C. Jun Y. Laulin J. P. Larcher A. Reynier P. (2000). Long-lasting hyperalgesia induced by fentanyl in rats: preventive effect of ketamine. *Anesthesiology* 92 465–472. 10.1097/00000542-200002000-00029 10691234

[B58] Center for Behavioral Health Statistics and Quality (2019). *SAMHSA, National Survey on Drug Use and Health. Key Substance Use and Mental Health Indicators in the United States: Results from the 2018 National Survey on Drug Use and Health.* Rockville, MD: HHS Public.

[B59] Chartoff E. H. Mavrikaki M. (2015). Sex differences in kappa opioid receptor function and their potential impact on addiction. *Front. Neurosci.* 9:466. 10.3389/fnins.2015.00466 26733781 PMC4679873

[B60] Chefer V. I. Ba C. M. (2013). Kappa opioid receptors on dopaminergic neurons are necessary for kappa-mediated place aversion. *Neuropsychopharmacology* 38 2623–2631. 10.1038/npp.2013.171 23921954 PMC3828533

[B61] Chefer V. I. Shippenberg T. S. (2009). Augmentation of morphine-induced sensitization but reduction in morphine tolerance and reward in delta-opioid receptor knockout mice. *Neuropsychopharmacology* 34 887–898. 10.1038/npp.2008.128 18704097 PMC2639630

[B62] Chen Y. Mestek A. Liu J. Hurley J. A. Yu L. (1993). Molecular cloning and functional expression of a mu-opioid receptor from rat brain. *Mol. Pharmacol.* 44 8–12. 8393525

[B63] Chou R. Turner J. A. Devine E. B. Hansen R. N. Sullivan S. D. Blazina I. (2015). The effectiveness and risks of long-term opioid therapy for chronic pain: a systematic review for a national institutes of health pathways to prevention workshop. *Am. College Phys.* 162 276–286. 10.7326/M14-2559 25581257

[B64] Chu L. F. Angst M. S. Clark D. (2008). Opioid-induced hyperalgesia in humans: molecular mechanisms and clinical considerations. *Clin. J. Pain* 24 479–496. 10.1097/AJP.0b013e31816b2f43 18574358

[B65] Chu Sin Chung P. Kieffer B. L. (2013). Delta opioid receptors in brain function and diseases. *Pharmacol. Ther.* 140 112–120. 10.1016/J.PHARMTHERA.2013.06.003 23764370 PMC3775961

[B66] Chung C. P. Dupont W. D. Murray K. T. Hall K. Stein C. M. Ray W. A. (2019). Comparative out-of-hospital mortality of long-acting opioids prescribed for non-cancer pain: a retrospective cohort study. *Pharmacoepidemiol. Drug Saf.* 28 48–53. 10.1002/pds.4619 30003613 PMC6330158

[B67] Chung S. Pohl S. Zeng J. Civelli O. Reinscheid R. K. (2006). Endogenous orphanin FQ/nociceptin is involved in the development of morphine tolerance. *J. Pharmacol. Exp. Ther.* 318 262–267. 10.1124/jpet.106.103960 16595734

[B68] Ciccocioppo R. Angeletti S. Sanna P. P. Weiss F. Massi M. (2000). Effect of nociceptin/orphanin FQ on the rewarding properties of morphine. *Eur. J. Pharmacol.* 404 153–159. 10.1016/S0014-2999(00)00590-2 10980274

[B69] Ciccocioppo R. Economidou D. Fedeli A. Angeletti S. Weiss F. Heilig M. (2004). Attenuation of ethanol self-administration and of conditioned reinstatement of alcohol-seeking behaviour by the antiopioid peptide nociceptin/orphanin FQ in alcohol-preferring rats. *Psychopharmacology* 172 170–178. 10.1007/S00213-003-1645-1 14624331 PMC3035816

[B70] Ciccocioppo R. Panocka I. Polidori C. Regoli D. Massi M. (1999). Effect of nociceptin on alcohol intake in alcohol-preferring rats. *Psychopharmacology* 141 220–224. 10.1007/S002130050828 9952048

[B71] Cicero T. J. Ellis M. S. Chilcoat H. D. (2018). Understanding the use of diverted buprenorphine. *Drug Alcohol Depend.* 193 117–123. 10.1016/J.DRUGALCDEP.2018.09.007 30359928

[B72] Clarke T. K. Ambrose-Lanci L. Ferraro T. N. Berrettini W. H. Kampman K. M. Dackis C. A. (2012). Genetic association analyses of PDYN polymorphisms with heroin and cocaine addiction. *Genes Brain Behav.* 11 415–423. 10.1111/J.1601-183X.2012.00785.X 22443215 PMC11987048

[B73] Colpaert F. C. Meert T. de Witte P. Schmitt P. (1982). Further evidence validating adjuvant arthritis as an experimental model of chronic pain in the rat. *Life Sci.* 31 67–75. 10.1016/0024-3205(82)90402-7 7109855

[B74] Colpaert F. C. Tarayre J. P. Alliaga M. Bruins Slot L. A. Attal N. Koek W. (2001). Opiate self-administration as a measure of chronic nociceptive pain in arthritic rats. *Pain* 91 33–45. 10.1016/S0304-3959(00)00413-9 11240076

[B75] Compton W. M. Volkow N. D. (2006). Major increases in opioid analgesic abuse in the United States: concerns and strategies. *Drug Alcohol Depend.* 81 103–107. 10.1016/j.drugalcdep.2005.05.009 16023304

[B76] Cong X. Maurel D. Déméné H. Vasiliauskaité-Brooks I. Hagelberger J. Peysson F. (2021). Molecular insights into the biased signaling mechanism of the μ-opioid receptor. *Mol. Cell* 81 4165.e6–4175.e6. 10.1016/j.molcel.2021.07.033 34433090 PMC8541911

[B77] Contet C. Kieffer B. L. Befort K. (2004). Mu opioid receptor: a gateway to drug addiction. *Curr. Opin. Neurobiol.* 14 370–378. 10.1016/J.CONB.2004.05.005 15194118

[B78] Conway S. M. Puttick D. Russell S. Potter D. Roitman M. F. Chartoff E. H. (2019). Females are less sensitive than males to the motivational- and dopamine-suppressing effects of kappa opioid receptor activation. *Neuropharmacology* 146 231–241. 30528327 10.1016/j.neuropharm.2018.12.002 PMC6339824

[B79] Corder G. Castro D. C. Bruchas M. R. Scherrer G. (2018). Endogenous and exogenous opioids in pain. *Annu. Rev. Neurosci.* 41 453–473. 10.1146/annurev-neuro-080317-061522 29852083 PMC6428583

[B80] Corder G. Tawfik V. L. Wang D. Sypek E. I. Low S. A. Dickinson J. R. (2017). Loss of μ opioid receptor signaling in nociceptors, but not microglia, abrogates morphine tolerance without disrupting analgesia. *Nat. Med.* 23 164–173. 10.1038/nm.4262 28092666 PMC5296291

[B81] Cork R. Hameroff S. Weiss J. (1985). Effects of halothane and fentanyl anesthesia on plasma β-endorphin immunoreactivity during cardiac surgery. *Anesth. Analg.* 64 677–680. 3160261

[B82] Cowan A. Lewis J. W. Macfarlane I. R. (1977). Agonist and antagonist properties of buprenorphine, a new antinociceptive agent. *Br. J. Pharmacol.* 60 537–545. 10.1111/j.1476-5381.1977.tb07532.x 409448 PMC1667394

[B83] Cox B. M. Goldstein A. Li C. H. (1976). Opioid activity of a peptide, beta-lipotropin-(61-91), derived from beta-lipotropin. *Proc. Natl. Acad. Sci. U.S.A.* 73 1821–1823. 10.1073/PNAS.73.6.1821 1064855 PMC430398

[B84] Crist R. C. Ambrose-Lanci L. M. Vaswani M. Clarke T. K. Zeng A. Yuan C. (2013). Case-control association analysis of polymorphisms in the delta-opioid receptor, OPRD1, with cocaine and opioid addicted populations. *Drug Alcohol Depend.* 127 122–128. 10.1016/j.drugalcdep.2012.06.023 22795689 PMC3509227

[B85] Cui Y. Ostlund S. B. James A. S. Park C. S. Ge W. Roberts K. W. (2014). Targeted expression of μ-opioid receptors in a subset of striatal direct-pathway neurons restores opiate reward. *Nat. Neurosci.* 17 254–261. 10.1038/NN.3622 24413699 PMC4008330

[B86] Cunha A. M. Esteves M. Pereira-Mendes J. Guimarães M. R. Almeida A. Leite-Almeida H. (2020). High trait impulsivity potentiates the effects of chronic pain on impulsive behavior. *Neurobiol. Pain* 7:100042. 10.1016/J.YNPAI.2019.100042 31890992 PMC6928455

[B87] Dagnino A. P. A. da Silva R. B. M. Chagastelles P. C. Pereira T. C. B. Venturin G. T. Greggio S. (2019). Nociceptin/orphanin FQ receptor modulates painful and fatigue symptoms in a mouse model of fibromyalgia. *Pain* 160 1383–1401. 10.1097/J.PAIN.0000000000001513 30720581

[B88] Dahan A. van Dam C. J. Niesters M. van Velzen M. Fossler M. J. Demitrack M. A. (2020). Benefit and risk evaluation of biased μ-receptor agonist oliceridine versus morphine. *Anesthesiology* 133 559–568. 10.1097/ALN.0000000000003441 32788558

[B89] Darcq E. Kieffer B. L. (2018). Opioid receptors: drivers to addiction? *Nat. Rev. Neurosci.* 19 499–514. 10.1038/s41583-018-0028-x 29934561

[B90] DaSilva A. F. Nascimento T. D. DosSantos M. F. Lucas S. van Holsbeeck H. DeBoer M. (2014). μ-Opioid activation in the prefrontal cortex in migraine attacks – brief report I. *Ann. Clin. Transl. Neurol.* 1:439. 10.1002/ACN3.65 25072055 PMC4110741

[B91] David V. Matifas A. Gavello-Baudy S. Decorte L. Kieffer B. L. Cazala P. (2008). Brain regional Fos expression elicited by the activation of mu- but not delta-opioid receptors of the ventral tegmental area: evidence for an implication of the ventral thalamus in opiate reward. *Neuropsychopharmacology* 33 1746–1759. 10.1038/SJ.NPP.1301529 17895918

[B92] Davis A. M. Inturrisi C. E. (1999). d-Methadone blocks morphine tolerance and N-methyl-D-aspartate-induced hyperalgesia. *J. Pharmacol. Exp. Ther.* 289 1048–1053. 10215686

[B93] Davis M. A. Lin L. A. Liu H. Sites B. D. (2017). Prescription opioid use among adults with mental health disorders in the United States. *J. Am. Board Fam. Med.* 30 407–417. 10.3122/JABFM.2017.04.170112 28720623

[B94] DeLeo J. A. Tanga F. Y. Tawfik V. L. (2004). Neuroimmune activation and neuroinflammation in chronic pain and opioid tolerance/hyperalgesia. *Neuroscientist* 10 40–52. 10.1177/1073858403259950 14987447

[B95] Depner U. B. Reinscheid R. K. Takeshima H. Brune K. Zeilhofer H. U. (2003). Normal sensitivity to acute pain, but increased inflammatory hyperalgesia in mice lacking the nociceptin precursor polypeptide or the nociceptin receptor. *Eur. J. Neurosci.* 17 2381–2387. 10.1046/J.1460-9568.2003.02676.X 12814369

[B96] Derouiche L. Pierre F. Doridot S. Ory S. Massotte D. (2020). Heteromerization of endogenous mu and delta opioid receptors induces ligand-selective co-targeting to lysosomes. *Molecules* 25:4493. 10.3390/molecules25194493 33007971 PMC7583997

[B97] Devine D. P. Reinscheid R. K. Monsma F. J. Civelli O. Akil H. (1996). The novel neuropeptide orphanin FQ fails to produce conditioned place preference or aversion. *Brain Res.* 727 225–229. 10.1016/0006-8993(96)00476-3 8842403

[B98] Devine D. P. Wise R. A. (1994). Self-administration of morphine, DAMGO, and DPDPE into the ventral tegmental area of rats. *J. Neurosci.* 14 1978–1984. 10.1523/jneurosci.14-04-01978.1994 8158252 PMC6577111

[B99] DeWire S. M. Yamashita D. S. Rominger D. H. Liu G. Cowan C. L. Graczyk T. M. (2013). A G protein-biased ligand at the μ-opioid receptor is potently analgesic with reduced gastrointestinal and respiratory dysfunction compared with morphines. *J. Pharmacol. Exp. Ther.* 344 708–717. 10.1124/jpet.112.201616 23300227

[B100] Dickenson A. H. Navratilova E. Patel R. Porreca F. Bannister K. (2020). Supraspinal opioid circuits differentially modulate spinal neuronal responses in neuropathic rats. *Anesthesiology* 132 881–894. 10.1097/ALN.0000000000003120 31977518

[B101] Dickenson A. H. Sullivan A. F. McQuay H. J. (1990). Intrathecal etorphine, fentanyl and buprenorphine on spinal nociceptive neurones in the rat. *Pain* 42 227–234. 10.1016/0304-3959(90)91166-G 2247319

[B102] Dietis N. Rowbotham D. J. Lambert D. G. (2011). Opioid receptor subtypes: fact or artifact? *Br. J. Anaesth.* 107 8–18. 10.1093/BJA/AER115 21613279

[B103] Dole V. P. Nyswander M. (1965). A medical treatment for diacetylmorphine (Heroin) addiction: a clinical trial with methadone hydrochloride. *JAMA* 193 646–650. 10.1001/JAMA.1965.03090080008002 14321530

[B104] Dole V. P. Nyswander M. E. (1976). Methadone maintenance treatment: a ten-year perspective. *JAMA* 235 2117–2119. 10.1001/JAMA.1976.03260450029025 946538

[B105] Donica C. L. Awwad H. O. Thakker D. R. Standifer K. M. (2013). Cellular mechanisms of nociceptin/orphanin FQ (N/OFQ) Peptide (NOP) receptor regulation and heterologous regulation by N/OFQ. *Mol. Pharmacol.* 83 907–918. 10.1124/MOL.112.084632 23395957 PMC3629824

[B106] Donnelly C. R. Andriessen A. S. Chen G. Wang K. Jiang C. Maixner W. (2020). Central nervous system targets: glial cell mechanisms in chronic pain. *Neurotherapeutics* 17 846–860. 10.1007/S13311-020-00905-7 32820378 PMC7609632

[B107] DosSantos M. F. Martikainen I. K. Nascimento T. D. Love T. M. Deboer M. D. Maslowski E. C. (2012). Reduced basal ganglia μ-opioid receptor availability in trigeminal neuropathic pain: a pilot study. *Mol. Pain* 8:74. 10.1186/1744-8069-8-74 23006894 PMC3522528

[B108] DosSantos M. F. Moura B. de S. DaSilva A. F. (2017). Reward circuitry plasticity in pain perception and modulation. *Front. Pharmacol.* 8:790. 10.3389/fphar.2017.00790 29209204 PMC5702349

[B109] Doyle M. A. Mazei-Robison M. S. (2021). Opioid-induced molecular and cellular plasticity of ventral tegmental area dopamine neurons. *Cold Spring Harb. Perspect. Med.* 11 1–15. 10.1101/cshperspect.a039362 31964652 PMC7371531

[B110] Drdla R. Gassner M. Gingl E. Sandkühler J. (2009). Induction of synaptic long-term potentiation after opioid withdrawal. *Science* 325 207–210. 10.1126/SCIENCE.1171759/SUPPL_FILE/DRDLA.SOM.PDF 19590003

[B111] Drdla-Schutting R. Benrath J. Wunderbaldinger G. Sandkühler J. (2012). Erasure of a spinal memory trace of pain by a brief, high-dose opioid administration. *Science* 335 235–238. 10.1126/SCIENCE.1211726/SUPPL_FILE/DRDLA-SCHUTTING.SOM.PDF 22246779

[B112] Duarte D. F. (2005). Opium and opioids: a brief history. *Rev. Bras. Anestesiol.* 55 135–146. 10.1590/S0034-70942005000100015 19471817

[B113] Dubois M. Pickar D. Cohen M. Gadde P. Macnamara T. E. Bunney W. E. (1982). Effects of fentanyl on the response of plasma beta-endorphin immunoreactivity to surgery. *Anesthesiology* 57 468–472. 10.1097/00000542-198212000-00006 6293344

[B114] Dumas E. O. Pollack G. M. (2008). Opioid tolerance development: a pharmacokinetic/pharmacodynamic perspective. *AAPS J.* 10 537–551. 10.1208/s12248-008-9056-1 18989788 PMC2628209

[B115] Economidou D. Hansson A. C. Weiss F. Terasmaa A. Sommer W. H. Cippitelli A. (2008). Dysregulation of nociceptin/orphanin FQ activity in the amygdala is linked to excessive alcohol drinking in the rat. *Biol. Psychiatry* 64 211–218. 10.1016/j.biopsych.2008.02.004 18367152 PMC4275225

[B116] Eidson L. N. Inoue K. Young L. J. Tansey M. G. Murphy A. Z. (2016). Toll-like receptor 4 mediates morphine-induced neuroinflammation and tolerance via soluble tumor necrosis factor signaling. *Neuropsychopharmacology* 42 661–670. 10.1038/npp.2016.131 27461080 PMC5240168

[B117] Eidson L. N. Murphy A. Z. (2019). Inflammatory mediators of opioid tolerance: implications for dependency and addiction. *Peptides* 115 51–58. 10.1016/j.peptides.2019.01.003 30890355 PMC6863079

[B118] Eikemo M. Løseth G. E. Leknes S. (2021). Do endogenous opioids mediate or fine-tune human pain relief? *Pain* 162 2789–2791. 10.1097/j.pain.0000000000002286 34793404

[B119] Elman I. Borsook D. (2016). Common brain mechanisms of chronic pain and addiction. *Neuron* 89 11–36. 10.1016/j.neuron.2015.11.027 26748087

[B120] Emery M. A. Akil H. (2020). Endogenous opioids at the intersection of opioid addiction, pain, and depression: the search for a precision medicine approach. *Annu. Rev. Neurosci.* 43 355–374. 10.1146/annurev-neuro-110719-095912 32109184 PMC7646290

[B121] Escobar A. del P. Casanova J. P. Andrés M. E. Fuentealba J. A. (2020). Crosstalk between kappa opioid and dopamine systems in compulsive behaviors. *Front. Pharmacol.* 11:57. 10.3389/FPHAR.2020.00057/BIBTEX PMC7040183 32132923

[B122] Evans C. J. Keith D. E. Morrison H. Magendzo K. Edwards R. H. (1992). Cloning of a delta opioid receptor by functional expression. *Science* 258 1952–1955. 10.1126/science.1335167 1335167

[B123] Ewan E. E. Martin T. J. (2011). Opioid facilitation of rewarding electrical brain stimulation is suppressed in rats with neuropathic pain. *Anesthesiology* 114 624–632. 10.1097/ALN.0b013e31820a4edb 21293250 PMC3073684

[B124] Fallon N. Brown C. Twiddy H. Brian E. Frank B. Nurmikko T. (2021). Adverse effects of COVID-19-related lockdown on pain, physical activity and psychological well-being in people with chronic pain. *Br. J. Pain* 15 357–368. 10.1177/2049463720973703 34377461 PMC8339954

[B125] Ferrari A. Coccia C. P. R. Bertolini A. Sternieri E. (2004). Methadone—metabolism, pharmacokinetics and interactions. *Pharmacol. Res.* 50 551–559. 10.1016/J.PHRS.2004.05.002 15501692

[B126] Fibiger H. C. LePiane F. G. Jakubovic A. Phillips A. G. (1987). The role of dopamine in intracranial self-stimulation of the ventral tegmental area. *J. Neurosci.* 7 3888–3896. 10.1523/jneurosci.07-12-03888.1987 3121802 PMC6569118

[B127] Filliol D. Ghozland S. Chluba J. Martin M. Matthes H. W. D. Simonin F. (2000). Mice deficient for δ- and μ-opioid receptors exhibit opposing alterations of emotional responses. *Nat. Genet.* 25 195–200. 10.1038/76061 10835636

[B128] Florence C. Luo F. Rice K. (2021). The economic burden of opioid use disorder and fatal opioid overdose in the United States, 2017. *Drug Alcohol Depend.* 218:108350. 10.1016/j.drugalcdep.2020.108350 33121867 PMC8091480

[B129] Florin S. Meunier J. C. Costentin J. (2000). Autoradiographic localization of [3H]nociceptin binding sites in the rat brain. *Brain Res.* 880 11–16. 10.1016/S0006-8993(00)02669-X 11032985

[B130] Frank S. Veit R. Sauer H. Enck P. Friederich H. C. Unholzer T. (2016). Dopamine depletion reduces food-related reward activity independent of BMI. *Neuropsychopharmacology* 41 1551–1559. 10.1038/npp.2015.313 26450814 PMC4832016

[B131] Friedrich A. E. Gebhart G. F. (2003). Modulation of visceral hyperalgesia by morphine and cholecystokinin from the rat rostroventral medial medulla. *Pain* 104 93–101. 10.1016/S0304-3959(02)00469-4 12855318

[B132] Galaj E. Han X. Shen H. Jordan C. J. He Y. Humburg B. (2020). Dissecting the role of GABA neurons in the VTA versus SNr in opioid reward. *J. Neurosci.* 40 8853–8869. 10.1523/JNEUROSCI.0988-20.2020 33046548 PMC7659457

[B133] Gaskin D. J. Richard P. (2012). The economic costs of pain in the United States. *J. Pain* 13 715–724. 10.1016/J.JPAIN.2012.03.009 22607834

[B134] Gavériaux-Ruff C. Karchewski L. A. Hever X. Matifas A. Kieffer B. L. (2008). Inflammatory pain is enhanced in delta opioid receptor-knockout mice. *Eur. J. Neurosci.* 27 2558–2567. 10.1111/J.1460-9568.2008.06223.X 18513322 PMC4445739

[B135] Gaveriaux-Ruff C. Nozaki C. Nadal X. Hever X. C. Weibel R. Matifas A. (2011). Genetic ablation of delta opioid receptors in nociceptive sensory neurons increases chronic pain and abolishes opioid analgesia. *Pain* 152 1238–1248. 10.1016/J.PAIN.2010.12.031 21295407

[B136] Gendron L. Esdaile M. J. Mennicken F. Pan H. O’Donnell D. Vincent J. P. (2007a). Morphine priming in rats with chronic inflammation reveals a dichotomy between antihyperalgesic and antinociceptive properties of deltorphin. *Neuroscience* 144 263–274. 10.1016/J.NEUROSCIENCE.2006.08.077 17055663

[B137] Gendron L. Pintar J. E. Chavkin C. (2007b). Essential role of mu opioid receptor in the regulation of delta opioid receptor-mediated antihyperalgesia. *Neuroscience* 150 807–817. 10.1016/J.NEUROSCIENCE.2007.09.060 17997230 PMC2194644

[B138] Gendron L. Lucido A. L. Mennicken F. O’Donnell D. Vincent J. P. Stroh T. (2006). Morphine and pain-related stimuli enhance cell surface availability of somatic δ-opioid receptors in rat dorsal root ganglia. *J. Neurosci.* 26 953–962. 10.1523/JNEUROSCI.3598-05.2006 16421315 PMC6675352

[B139] Ghozland S. Matthes H. W. D. Simonin F. Filliol D. Kieffer B. L. Maldonado R. (2002). Motivational effects of cannabinoids are mediated by μ-opioid and κ-opioid receptors. *J. Neurosci.* 22 1146–1154. 10.1523/jneurosci.22-03-01146.2002 11826143 PMC6758535

[B140] Goldstick J. E. Guy G. P. Losby J. L. Baldwin G. Myers M. Bohnert A. S. B. (2021). Changes in initial opioid prescribing practices after the 2016 release of the CDC guideline for prescribing opioids for chronic pain. *JAMA Netw. Open* 4:e2116860. 10.1001/JAMANETWORKOPEN.2021.16860 34255047 PMC8278262

[B141] Gomes I. Gupta A. Filipovska J. Szeto H. H. Pintar J. E. Devi L. A. (2004). A role for heterodimerization of mu and delta opiate receptors in enhancing morphine analgesia. *Proc. Natl. Acad. Sci. U.S.A.* 101 5135–5139. 10.1073/PNAS.0307601101 15044695 PMC387386

[B142] Graeme Henderson C. Hill R. Disney A. Conibear A. Sutcliffe K. Dewey W. (2018). The novel μ-opioid receptor agonist PZM21 depresses respiration and induces tolerance to antinociception. *Br. J. Pharmacol.* 175:2653. 10.1111/bph.14224 29582414 PMC6003631

[B143] Grol-Prokopczyk H. (2017). Sociodemographic disparities in chronic pain, based on 12-year longitudinal data. *Pain* 158 313–322. 10.1097/J.PAIN.0000000000000762 28092650 PMC5242384

[B144] Gudin J. Fudin J. (2020). A narrative pharmacological review of buprenorphine: a unique opioid for the treatment of chronic pain. *Pain Ther.* 9 41–54. 10.1007/S40122-019-00143-6/TABLES/2 31994020 PMC7203271

[B145] Hagelberg N. Aalto S. Tuominen L. Pesonen U. Någren K. Hietala J. (2012). Striatal μ-opioid receptor availability predicts cold pressor pain threshold in healthy human subjects. *Neurosci. Lett.* 521 11–14. 10.1016/J.NEULET.2012.05.042 22622175

[B146] Halbert B. T. Davis R. B. Wee C. C. (2016). Disproportionate longer-term opioid use among US adults with mood disorders. *Pain* 157:2452. 10.1097/J.PAIN.0000000000000650 27472400 PMC5069117

[B147] Hans B. Kosterlitz H. Hughes J. (1977). Peptides with morphine-like action in the brain. *Br. J. Psychiatry* 130 298–304. 10.1192/BJP.130.3.298 14762

[B148] Hao J. X. Xu I. S. Wiesenfeld-Hallin Z. Xu X. J. (1998). Anti-hyperalgesic and anti-allodynic effects of intrathecal nociceptin/orphanin FQ in rats after spinal cord injury, peripheral nerve injury and inflammation. *Pain* 76 385–393. 10.1016/S0304-3959(98)00071-2 9718257

[B149] Hargreaves K. M. Dionne R. A. Mueller G. P. Goldstein D. S. Dubner R. (1986). Naloxone, fentanyl, and diazepam modify plasma beta-endorphin levels during surgery. *Clin. Pharmacol. Ther.* 40 165–171. 10.1038/CLPT.1986.159 3731680

[B150] Harris R. E. Clauw D. J. Scott D. J. Mclean S. A. Gracely R. H. Zubieta J.-K. (2007). Neurobiology of disease decreased central-opioid receptor availability in fibromyalgia. *J. Neurosci.* 27 10000–10006. 10.1523/JNEUROSCI.2849-07.2007 17855614 PMC6672650

[B151] Hassan A. H. S. Ableitner A. Stein C. Herz A. (1993). Inflammation of the rat paw enhances axonal transport of opioid receptors in the sciatic nerve and increases their density in the inflamed tissue. *Neuroscience* 55 185–195. 10.1016/0306-4522(93)90465-R 7688879

[B152] Hay J. L. White J. M. Bochner F. Somogyi A. A. Semple T. J. Rounsefell B. (2009). Hyperalgesia in opioid-managed chronic pain and opioid-dependent patients. *J. Pain* 10 316–322. 10.1016/J.JPAIN.2008.10.003 19101210

[B153] Hayes A. G. Stewart B. R. (1985). Effect of μ and κ opioid receptor agonists on rat plasma corticosterone levels. *Eur. J. Pharmacol.* 116 75–79. 10.1016/0014-2999(85)90186-4 2996912

[B154] Hayes C. Krebs E. Hudson T. Brown J. Li C. Martin B. (2019). PMS31 impact of opioid dose escalation on the development of substance use disorders, accidents, self-inflicted injuries, opioid overdoses, and alcohol and non-opioid drug-related overdoses: a retrospective cohort study. *Value Health* 22:S244. 10.1016/j.jval.2019.04.1141 PMC7263736 31944486

[B155] Hayes C. J. Krebs E. E. Hudson T. Brown J. Li C. Martin B. C. (2020). Impact of opioid dose escalation on pain intensity. *Pain* 161 979–988. 10.1097/j.pain.0000000000001784 31917775 PMC7510136

[B156] Hayhurst C. J. Durieux M. E. (2016). Differential opioid tolerance and opioid-induced hyperalgesiaa clinical reality. *Anesthesiology* 124 483–488. 10.1097/ALN.0000000000000963 26594912

[B157] Heinl C. Drdla-Schutting R. Xanthos D. N. Sandkühler J. (2011). Distinct mechanisms underlying pronociceptive effects of opioids. *J. Neurosci.* 31 16748–16756. 10.1523/JNEUROSCI.3491-11.2011 22090501 PMC6633307

[B158] Heinricher M. M. Neubert M. J. (2004). Neural basis for the hyperalgesic action of cholecystokinin in the rostral ventromedial medulla. *J. Neurophysiol.* 92 1982–1989. 10.1152/JN.00411.2004/ASSET/IMAGES/LARGE/Z9K0100441300007.JPEG 15152023

[B159] Hertz S. (2018). *FDA Briefing Document Anesthetic and Analgesic Drug Products Advisory Committee (AADPAC) Meeting.* Silver Spring, MD: Food and Drug Administration, 1–115.

[B160] Higgins C. Smith B. H. Matthews K. (2020). Comparison of psychiatric comorbidity in treatment-seeking, opioid-dependent patients with versus without chronic pain. *Addiction* 115 249–258. 10.1111/ADD.14768 31386238

[B161] Hipólito L. Sánchez-Catalán M. J. Zanolini I. Polache A. Granero L. (2008). Shell/core differences in mu- and delta-opioid receptor modulation of dopamine efflux in nucleus accumbens. *Neuropharmacology* 55 183–189. 10.1016/J.NEUROPHARM.2008.05.012 18582908

[B162] Hipólito L. Wilson-Poe A. Campos-Jurado Y. Zhong E. Gonzalez-Romero J. Virag L. (2015). Inflammatory pain promotes increased opioid self-administration: role of dysregulated ventral tegmental area μ opioid receptors. *J. Neurosci.* 35 12217–12231. 10.1523/JNEUROSCI.1053-15.2015 26338332 PMC4556787

[B163] Hirose N. Murakawa K. Takada K. Oi Y. Suzuki T. Nagase H. (2005). Interactions among mu- and delta-opioid receptors, especially putative delta1- and delta2-opioid receptors, promote dopamine release in the nucleus accumbens. *Neuroscience* 135 213–225. 10.1016/J.NEUROSCIENCE.2005.03.065 16111831

[B164] Højsted J. Sjøgren P. (2007). Addiction to opioids in chronic pain patients: a literature review. *Eur. J. Pain* 11 490–518. 10.1016/J.EJPAIN.2006.08.004 17070082

[B165] Horikawa S. Takai T. Toyosato M. Takahashi H. Noda M. Kakidani H. (1983). Isolation and structural organization of the human preproenkephalin Bgene. *Nature* 306 611–614. 10.1038/306611a0 6316163

[B166] Hser Y. I. Mooney L. J. Saxon A. J. Miotto K. Bell D. S. Huang D. (2017). Chronic pain among patients with opioid use disorder: results from electronic health records data. *J. Subst. Abuse Treat.* 77 26–30. 10.1016/J.JSAT.2017.03.006 28476267 PMC5424616

[B167] Hughes J. Kosterlitz H. W. Smith T. W. (1977). The distribution of methionin-enkephalin in the brain and peripheral tissues. *Br. J. Pharmacol.* 61 639–647. 10.1111/j.1476-5381.1977.tb07557.x 597668 PMC1668063

[B168] Huhn A. S. Berry M. S. Dunn K. E. (2018). Systematic review of sex-based differences in opioid-based effects. *Int. Rev. Psychiatry* 30 107–116. 10.1080/09540261.2018.1514295 30522374 PMC6551331

[B169] Hurd Y. L. Herman M. M. Hyde T. M. Bigelow L. B. Weinberger D. R. Kleinman J. E. (1997). Prodynorphin mRNA expression is increased in the patch vs matrix compartment of the caudate nucleus in suicide subjects. *Mol. Psychiatry* 2 495–500. 10.1038/SJ.MP.4000319 9399695

[B170] Hutchings J. B. Crampton D. Morris S. L. Durand D. Steinbring E. Combes F. (1997). *References and Notes Enhanced Morphine Analgesia in Mice Lacking-Arrestin 2.* Available online at: https://www.science.org (accessed April 24, 2022).

[B171] Iadarola M. J. Brady L. S. Draisci G. Dubner R. (1988). Enhancement of dynorphin gene expression in spinal cord following experimental inflammation: stimulus specificity, behavioral parameters and opioid receptor binding. *Pain* 35 313–326. 10.1016/0304-3959(88)90141-8 2906426

[B172] Ikeda K. Kobayashi T. Kumanishi T. Niki H. Yano R. (2000). Involvement of G-protein-activated inwardly rectifying K+ (GIRK) channels in opioid-induced analgesia. *Neurosci. Res.* 38 113–116. 10.1016/S0168-0102(00)00144-9 10997585

[B173] Institute of Medicine Committee on Advancing Pain Research (2011). *Relieving Pain in America: A Blueprint for Transforming Prevention, Care, Education, and Research.* Washington, DC: National Academies Press. 22553896

[B174] Iyengar S. Kim H. S. Wood P. L. (1986). Kappa opiate agonists modulate the hypothalamic-pituitary-adrenocortical axis in the rat. *J. Pharmacol. Exp. Ther.* 238 429–436. 3016237

[B175] Jantarada C. Silva C. Guimarães-Pereira L. (2021). Prevalence of problematic use of opioids in patients with chronic noncancer pain: a systematic review with meta-analysis. *Pain Pract.* 21 715–729. 10.1111/PAPR.13001 33528858

[B176] Jarcho J. M. Mayer E. A. Jiang Z. K. Feier N. A. London E. D. (2012). Pain, affective symptoms and cognitive deficits in patients with cerebral dopamine dysfunction. *Pain* 153 744–754. 10.1016/J.PAIN.2012.01.002 22386471 PMC3816524

[B177] Jassar H. Nascimento T. D. Kaciroti N. Dossantos M. F. Danciu T. Koeppe R. A. (2019). Impact of chronic migraine attacks and their severity on the endogenous μ-opioid neurotransmission in the limbic system. *Neuroimage Clin.* 23:101905. 10.1016/j.nicl.2019.101905 31279240 PMC6612052

[B178] Ji M. J. Yang J. Gao Z. Q. Zhang L. Liu C. (2021). The role of the kappa opioid system in comorbid pain and psychiatric disorders: function and implications. *Front. Neurosci.* 15:130. 10.3389/FNINS.2021.642493/BIBTEX PMC7943636 33716658

[B179] Jones C. M. McCance-Katz E. F. (2019). Co-occurring substance use and mental disorders among adults with opioid use disorder. *Drug Alcohol Depend.* 197 78–82. 10.1016/j.drugalcdep.2018.12.030 30784952

[B180] Jutkiewicz E. M. Torregrossa M. M. Sobczyk-Kojiro K. Mosberg H. I. Folk J. E. Rice K. C. (2006). Behavioral and neurobiological effects of the enkephalinase inhibitor RB101 relative to its antidepressant effects. *Eur. J. Pharmacol.* 531 151–159. 10.1016/j.ejphar.2005.12.002 16442521 PMC1828120

[B181] Kabli N. Cahill C. M. (2007). Anti-allodynic effects of peripheral delta opioid receptors in neuropathic pain. *Pain* 127 84–93. 10.1016/J.PAIN.2006.08.003 16963185

[B182] Kallupi M. G Carrette L. L. Kononoff J. Solberg Woods L. C. Palmer A. A. Schweitzer P. (2020). Nociceptin attenuates the escalation of oxycodone self-administration by normalizing CeA–GABA transmission in highly addicted rats. *Natl. Acad. Sci.* 117 2140–2148. 10.1073/pnas.1915143117/-/DCSupplemental 31932450 PMC6994987

[B183] Kaplan H. Fields H. L. (1991). Hyperalgesia during acute opioid abstinence: evidence for a nociceptive facilitating function of the rostral ventromedial medulla. *J. Neurosci.* 11 1433–1439. 10.1523/jneurosci.11-05-01433.1991 2027054 PMC6575327

[B184] Kaplovitch E. Gomes T. Camacho X. Dhalla I. A. Mamdani M. M. Juurlink D. N. (2015). Sex differences in dose escalation and overdose death during chronic opioid therapy: a population-based cohort study. *PLoS One* 10:e0134550. 10.1371/journal.pone.0134550 26291716 PMC4546305

[B185] Karp J. F. Butters M. A. Begley A. E. Miller M. D. Lenze E. J. Blumberger D. M. (2014). Safety, tolerability, and clinical effect of low-dose buprenorphine for treatment-resistant depression in midlife and older adults. *J. Clin. Psychiatry* 75 e785–e793. 10.4088/JCP.13M08725 25191915 PMC4157317

[B186] Kathmann M. Flau K. Redmer A. Tränkle C. Schlicker E. (2006). Cannabidiol is an allosteric modulator at mu- and delta-opioid receptors. *Naunyn. Schmiedebergs Arch. Pharmacol.* 372 354–361. 10.1007/S00210-006-0033-X 16489449

[B187] Kendler K. S. Jacobson K. C. Prescott C. A. Neale M. C. (2003). Specificity of genetic and environmental risk factors for use and abuse/dependence of cannabis, cocaine, hallucinogens, sedatives, stimulants, and opiates in male twins. *Am. J. Psychiatry* 160 687–695. 10.1176/APPI.AJP.160.4.687 12668357

[B188] Kieffer B. Befort K. Gaveriaux-Ruff C. Hirth C. (1992). The delta-opioid receptor: isolation of a cDNA by expression cloning and pharmacological characterization. *Proc. Natl. Acad. Sci. U.S.A.* 89 12048–12052. 10.1073/PNAS.89.24.12048 1334555 PMC50695

[B189] Kliewer A. Gillis A. Hill R. Schmiedel F. Bailey C. Kelly E. (2020). Morphine-induced respiratory depression is independent of β-arrestin2 signalling. *Br. J. Pharmacol.* 177 2923–2931. 10.1111/BPH.15004 32052419 PMC7280004

[B190] Kliewer A. Schmiedel F. Sianati S. Bailey A. Bateman J. T. Levitt E. S. (2019). Phosphorylation-deficient G-protein-biased μ-opioid receptors improve analgesia and diminish tolerance but worsen opioid side effects. *Nat. Commun.* 10 1–11. 10.1038/s41467-018-08162-1 30664663 PMC6341117

[B191] Ko M. C. Naughton N. N. (2009). Antinociceptive effects of nociceptin/orphanin FQ administered intrathecally in monkeys. *J. Pain* 10 509–516. 10.1016/j.jpain.2008.11.006 19231294 PMC2797530

[B192] Koch T. Höllt V. (2008). Role of receptor internalization in opioid tolerance and dependence. *Pharmacol. Ther.* 117 199–206. 10.1016/J.PHARMTHERA.2007.10.003 18076994

[B193] Koob G. F. (2020). Neurobiology of opioid addiction: opponent process, hyperkatifeia, and negative reinforcement. *Biol. Psychiatry* 87 44–53. 10.1016/j.biopsych.2019.05.023 31400808

[B194] Koob G. F. Volkow N. D. (2010). Neurocircuitry of addiction. *Neuropsychopharmacology* 35 217–238. 10.1038/npp.2010.4 19710631 PMC2805560

[B195] Koppert W. Ihmsen H. Körber N. Wehrfritz A. Sittl R. Schmelz M. (2005). Different profiles of buprenorphine-induced analgesia and antihyperalgesia in a human pain model. *Pain* 118 15–22. 10.1016/J.PAIN.2005.06.030 16154698

[B196] Kotlinska J. Rafalski P. Biala G. Dylag T. Rolka K. Silberring J. (2003). Nociceptin inhibits acquisition of amphetamine-induced place preference and sensitization to stereotypy in rats. *Eur. J. Pharmacol.* 474 233–239. 10.1016/S0014-2999(03)02081-8 12921868

[B197] Kotliñska J. Wichmann J. Legowska A. Rolka K. Silberring J. (2002). Orphanin FQ/nociceptin but not Ro 65-6570 inhibits the expression of cocaine-induced conditioned place preference. *Behav. Pharmacol.* 13 229–235. 10.1097/00008877-200205000-00006 12122313

[B198] Kovelowski C. J. Ossipov M. H. Sun H. Lai J. Malan T. P. Porreca F. (2000). Supraspinal cholecystokinin may drive tonic descending facilitation mechanisms to maintain neuropathic pain in the rat. *Pain* 87 265–273. 10.1016/s0304-3959(00)00290-6 10963906

[B199] Kreek M. J. (1973). Medical safety and side effects of methadone in tolerant individuals. *JAMA* 223 665–668. 10.1001/jama.1973.03220060039009 4739193

[B200] Kreek M. J. (1991). *Using Methadone Effectively: Achieving Goals by Application of Laboratory, Clinical, and Evaluation Research and by Development of Innovative Programs.* Bethesda, MD: NIDA Research Monograph Series, 245–266. 1922290

[B201] Kreek M. J. (2000). Methadone-related opioid agonist pharmacotherapy for heroin addiction. History, recent molecular and neurochemical research and future in mainstream medicine. *Ann. N.Y. Acad. Sci.* 909 186–216. 10.1111/j.1749-6632.2000.tb06683.x 10911931

[B202] Kreek M. J. Borg L. Ducat E. Ray B. (2010). Pharmacotherapy in the treatment of addiction: methadone. *J. Addict. Dis.* 29 200–216. 10.1080/10550881003684798 20407977 PMC2885886

[B203] Krishnamurti C. Rao S. C. (2016). The isolation of morphine by Serturner. *Indian J. Anaesth.* 60:861. 10.4103/0019-5049.193696 27942064 PMC5125194

[B204] Kuzmin A. Sandin J. Terenius L. Ögren S. O. (2004). Evidence in locomotion test for the functional heterogeneity of ORL-1 receptors. *Br. J. Pharmacol.* 141 132–140. 10.1038/sj.bjp.0705583 14662736 PMC1574169

[B205] Lane D. A. Tortorici V. Morgan M. M. (2004). Behavioral and electrophysiological evidence for tolerance to continuous morphine administration into the ventrolateral periaqueductal gray. *Neuroscience* 125 63–69. 10.1016/J.NEUROSCIENCE.2004.01.023 15051146

[B206] Lappin R. (2016). CDC issues new opioid prescribing guideline. *Emerg. Med.* 48 150–151. 10.15585/mmwr.rr6501e1er

[B207] Latif Z. E. H. Skjærvø I. Solli K. K. Tanum L. (2021). Chronic pain among patients with an opioid use disorder. *Am. J. Addict.* 30 366–375. 10.1111/ajad.13153 33738870

[B208] Laurent V. Bertran-Gonzalez J. Chieng B. C. Balleine B. W. (2014). δ-Opioid and dopaminergic processes in accumbens shell modulate the cholinergic control of predictive learning and choice. *J. Neurosci.* 34 1358–1369. 10.1523/JNEUROSCI.4592-13.2014 24453326 PMC3898294

[B209] Laurent V. Leung B. Maidment N. Balleine B. W. (2012). μ- and δ-opioid-related processes in the accumbens core and shell differentially mediate the influence of reward-guided and stimulus-guided decisions on choice. *J. Neurosci.* 32 1875–1883. 10.1523/JNEUROSCI.4688-11.2012 22302826 PMC3742880

[B210] le Merrer J. Becker J. A. J. Befort K. Kieffer B. L. (2009). Reward processing by the opioid system in the brain. *Physiol. Rev.* 89 1379–1412. 10.1152/physrev.00005.2009 19789384 PMC4482114

[B211] le Merrer J. Plaza-Zabala A. del Boca C. Matifas A. Maldonado R. Kieffer B. L. (2011). Deletion of the δ opioid receptor gene impairs place conditioning but preserves morphine reinforcement. *Biol. Psychiatry* 69 700–703. 10.1016/J.BIOPSYCH.2010.10.021 21168121

[B212] Lee M. C. Tracey I. (2010). Unravelling the mystery of pain, suffering, and relief with brain imaging. *Curr. Pain Headache Rep.* 14 124–131. 10.1007/S11916-010-0103-0 20425201

[B213] Lee M. O. Lee M. O. Silverman S. Hansen H. Patel V. Manchikanti L. (2011). A comprehensive review of opioid-induced hyperalgesia. *Pain Phys.* 14 145–161. 21412369

[B214] Leknes S. Lee M. Berna C. Andersson J. Tracey I. (2011). Relief as a reward: hedonic and neural responses to safety from pain. *PLoS One* 6:e17870. 10.1371/JOURNAL.PONE.0017870 21490964 PMC3072382

[B215] Leknes S. Tracey I. (2008). A common neurobiology for pain and pleasure. *Nat. Rev. Neurosci.* 9 314–320. 10.1038/NRN2333 18354400

[B216] Liang L. Lutz B. M. Bekker A. Tao Y. X. (2015). Epigenetic regulation of chronic pain. *Epigenomics* 7 235–242. 10.2217/EPI.14.75 25942533 PMC4422180

[B217] Liang Y. Chu H. Jiang Y. Yuan L. (2016). Morphine enhances IL-1β release through toll-like receptor 4-mediated endocytic pathway in microglia. *Purinergic Signal* 12 637–645. 10.1007/S11302-016-9525-4/FIGURES/7 27506813 PMC5124002

[B218] Ling G. S. F. Paul D. Simantov R. Pasternak G. W. (1989). Differential development of acute tolerance to analgesia, respiratory depression, gastrointestinal transit and hormone release in a morphine infusion model. *Life Sci.* 45 1627–1636. 10.1016/0024-3205(89)90272-5 2555641

[B219] Linz K. Christoph T. Tzschentke T. M. Koch T. Schiene K. Gautrois M. (2014). Cebranopadol: a novel potent analgesic nociceptin/orphanin FQ peptide and opioid receptor agonist. *J. Pharmacol. Exp. Ther.* 349 535–548. 10.1124/JPET.114.213694 24713140

[B220] Liu S. Pickens S. Burma N. E. Ibarra-Lecue I. Yang H. Xue L. (2019). Neurobiology of disease kappa opioid receptors drive a tonic aversive component of chronic pain. *J. Neurosci.* 39 4162–4178. 10.1523/JNEUROSCI.0274-19.2019 30862664 PMC6529867

[B221] Livingston K. E. Traynor J. R. (2018). Allostery at opioid receptors: modulation with small molecule ligands. *Br. J. Pharmacol.* 175:2846. 10.1111/BPH.13823 28419415 PMC6016636

[B222] Llorca-Torralba M. Pilar-Cuéllar F. Bravo L. Bruzos-Cidon C. Torrecilla M. Mico J. A. (2019a). Opioid activity in the locus coeruleus is modulated by chronic neuropathic pain. *Mol. Neurobiol.* 56 4135–4150. 10.1007/s12035-018-1361-9 30284123

[B223] Llorca-Torralba M. Suárez-Pereira I. Bravo L. Camarena-Delgado C. Garcia-Partida J. A. Mico J. A. (2019b). Chemogenetic silencing of the locus coeruleus–basolateral amygdala pathway abolishes pain-induced anxiety and enhanced aversive learning in rats. *Biol. Psychiatry* 85 1021–1035. 10.1016/j.biopsych.2019.02.018 30987747

[B224] Lord J. A. H. Waterfield A. A. Hughes J. Kosterlitz H. W. (1977). Endogenous opioid peptides: multiple agonists and receptors. *Nature* 267 495–499. 195217 10.1038/267495a0

[B225] Loyd D. R. Morgan M. M. Murphy A. Z. (2008). Sexually dimorphic activation of the periaqueductal gray-rostral ventromedial medullary circuit during the development of tolerance to morphine in the rat. *Eur. J. Neurosci.* 27 1517–1524. 10.1111/J.1460-9568.2008.06100.X 18364026 PMC2821209

[B226] Lucas J. Connor E. Bose J. (2021). Back, lower limb, and upper limb pain among U.S. Adults, 2019. *NCHS Data Brief* [Epub ahead of print]. 10.15620/CDC:107894 34473621

[B227] Lueptow L. M. Fakira A. K. Bobeck E. N. (2018). The contribution of the descending pain modulatory pathway in opioid tolerance. *Front. Neurosci.* 12:886. 10.3389/fnins.2018.00886 30542261 PMC6278175

[B228] Lutfy K. Cowan A. (2004). Buprenorphine: a unique drug with complex pharmacology. *Curr. Neuropharmacol.* 2:395. 10.2174/1570159043359477 18997874 PMC2581407

[B229] Lutfy K. Do T. Maidment N. T. (2001a). Orphanin FQ/nociceptin attenuates motor stimulation and changes in nucleus accumbens extracellular dopamine induced by cocaine in rats. *Psychopharmacology* 154 1–7. 10.1007/s002130000609 11291998 PMC2288655

[B230] Lutfy K. Hossain S. M. Khaliq I. Maidment N. T. (2001b). Orphanin FQ/nociceptin attenuates the development of morphine tolerance in rats. *Br. J. Pharmacol.* 134 529–534. 10.1038/SJ.BJP.0704279 11588106 PMC1572978

[B231] Lyness W. H. Smith F. L. Heavner J. E. Iacono C. U. Garvin R. D. (1989). Morphine self-administration in the rat during adjuvant-induced arthritis. *Life Sci.* 45 2217–2224. 10.1016/0024-3205(89)90062-3 2601574

[B232] Macey T. A. Bobeck E. N. Hegarty D. M. Aicher S. A. Ingram S. L. Morgan M. M. (2009). Extracellular signal-regulated kinase 1/2 activation counteracts morphine tolerance in the periaqueductal gray of the rat. *J. Pharmacol. Exp. Ther.* 331 412–418. 10.1124/JPET.109.152157 19684256 PMC2775267

[B233] Macey T. A. Lowe J. D. Chavkin C. (2006). Mu Opioid Receptor Activation of ERK1/2 Is GRK3 and arrestin dependent in striatal neurons. *J. Biol. Chem.* 281 34515–34524. 10.1074/JBC.M604278200 16982618 PMC2104781

[B234] Maher D. P. Zhang Y. Ahmed S. Doshi T. Malarick C. Stabach K. (2017). Chronic opioid therapy modifies QST changes after ketamine infusion in chronic pain patients. *J. Pain* 18 1468–1475. 10.1016/J.JPAIN.2017.07.008 28802882 PMC5729746

[B235] Makary M. M. Polosecki P. Cecchi G. A. DeAraujo I. E. Barron D. S. Constable T. R. (2020). Loss of nucleus accumbens low-frequency fluctuations is a signature of chronic pain. *Proc. Natl. Acad. Sci. U.S.A.* 117 10015–10023. 10.1073/PNAS.1918682117 32312809 PMC7211984

[B236] Manchikanti L. Vanaparthy R. Atluri S. Sachdeva H. Kaye A. D. Hirsch J. A. (2021). COVID-19 and the opioid epidemic: two public health emergencies that intersect with chronic pain. *Pain Ther.* 10 269–286. 10.1007/S40122-021-00243-2 33718982 PMC7955940

[B237] Manglik A. Lin H. Aryal D. K. McCorvy J. D. Dengler D. Corder G. (2016). Structure-based discovery of opioid analgesics with reduced side effects. *Nature* 537 185–190. 10.1038/nature19112 27533032 PMC5161585

[B238] Manubay J. Davidson J. Vosburg S. Jones J. Comer S. Sullivan M. (2015). Sex differences among opioid-abusing chronic pain patients in a clinical trial. *J. Addict. Med.* 9:46. 10.1097/ADM.0000000000000086 25325300 PMC4310755

[B239] Mao J. Sung B. Ji R.-R. Lim G. (2002). Chronic morphine induces downregulation of spinal glutamate transporters: implications in morphine tolerance and abnormal pain sensitivity. *J. Neurosci.* 22 8312–8323. 10.1523/JNEUROSCI.22-18-08312.2002 12223586 PMC6758088

[B240] Marchand S. (2008). The physiology of pain mechanisms: from the periphery to the brain. *Rheum. Dis. Clin. North Am.* 34 285–309. 10.1016/J.RDC.2008.04.003 18638678

[B241] Mark T. L. Parish W. (2019). Opioid medication discontinuation and risk of adverse opioid-related health care events. *J. Subst. Abuse Treat.* 103 58–63. 10.1016/j.jsat.2019.05.001 31079950

[B242] Markovic T. Pedersen C. E. Massaly N. Vachez Y. M. Ruyle B. Murphy C. A. (2021). Pain induces adaptations in ventral tegmental area dopamine neurons to drive anhedonia-like behavior. *Nat. Neurosci.* 24 1601–1613. 10.1038/s41593-021-00924-3 34663957 PMC8556343

[B243] Martikainen I. K. Nuechterlein E. B. Peciña M. Love T. M. Cummiford C. M. Green C. R. (2015). Chronic back pain is associated with alterations in dopamine neurotransmission in the ventral striatum. *J. Neurosci.* 35 9957–9965. 10.1523/JNEUROSCI.4605-14.2015 26156996 PMC4495244

[B244] Martikainen I. K. Peciña M. Love T. M. Nuechterlein E. B. Cummiford C. M. Green C. R. (2013). Alterations in endogenous opioid functional measures in chronic back pain. *J. Neurosci.* 33 14729–14737. 10.1523/JNEUROSCI.1400-13.2013 24027273 PMC3771036

[B245] Martin T. J. Kim S. A. Buechler N. L. Porreca F. Eisenach J. C. (2007). Opioid Self-administration in the nerve-injured ratrelevance of antiallodynic effects to drug consumption and effects of intrathecal analgesics. *Anesthesiology* 106 312–322. 10.1097/00000542-200702000-00020 17264726

[B246] Martin T. J. Kim S. A. Eisenach J. C. (2006). Clonidine maintains intrathecal self-administration in rats following spinal nerve ligation. *Pain* 125 257–263. 10.1016/J.PAIN.2006.05.027 16806709

[B247] Martin W. R. Eades C. G. Thompson J. A. Huppler R. E. Gilbert P. E. (1976). The effects of morphine and nalorphine like drugs in the nondependent and morphine dependent chronic spinal dog. *J. Pharmacol. Exp. Ther.* 197 517–532. 945347

[B248] Massaly N. Copits B. A. Wilson-Poe A. R. Hipólito L. Markovic T. Yoon H. J. (2019). Pain-induced negative affect is mediated via recruitment of the nucleus accumbens kappa opioid system. *Neuron* 102 564.e6–573.e6. 10.1016/j.neuron.2019.02.029 30878290 PMC6509001

[B249] Massaly N. Markovic T. Creed M. Al-Hasani R. Cahill C. M. Moron J. A. (2021). Pain, negative affective states and opioid-based analgesics: safer pain therapies to dampen addiction. *Int. Rev. Neurobiol.* 157 31–68. 10.1016/BS.IRN.2020.09.002 33648672

[B250] Massaly N. Morón J. A. (2019). Pain and opioid systems, implications in the opioid epidemic. *Curr. Opin. Behav. Sci.* 26 69–74. 10.1016/j.cobeha.2018.10.002 30984806 PMC6457459

[B251] Massaly N. Temp J. Machelska H. Stein C. (2020). Uncovering the analgesic effects of a pH-dependent mu-opioid receptor agonist using a model of nonevoked ongoing pain. *Pain* 161 2798–2804. 10.1097/J.PAIN.0000000000001968 32639370

[B252] Matsui A. Jarvie B. C. Robinson B. G. Hentges S. T. Williams J. T. (2014). Separate GABA afferents to dopamine neurons mediate acute action of opioids, development of tolerance, and expression of withdrawal. *Neuron* 82 1346–1356. 10.1016/J.NEURON.2014.04.030 24857021 PMC4072256

[B253] Mattick R. P. Breen C. Kimber J. Davoli M. (2009). Methadone maintenance therapy versus no opioid replacement therapy for opioid dependence. *Cochrane Database Syst. Rev.* 3:CD002209. 10.1002/14651858.CD002209.pub2 12519570

[B254] McHugh R. K. (2020). The importance of studying sex and gender differences in opioid misuse. *JAMA Netw. Open* 3:e2030676. 10.1001/JAMANETWORKOPEN.2020.30676 33331915

[B255] Meisenberg B. R. Grover J. Campbell C. Korpon D. (2018). Assessment of opioid prescribing practices before and after implementation of a health system intervention to reduce opioid overprescribing. *JAMA Netw. Open* 1:e182908. 10.1001/jamanetworkopen.2018.2908 30646184 PMC6324493

[B256] Meunier J. C. Mollereau C. Toll L. Suaudeau C. Moisand C. Alvinerie P. (1995). Isolation and structure of the endogenous agonist of opioid receptor-like ORL1 receptor. *Nature* 377 532–535. 10.1038/377532a0 7566152

[B257] Meyer P. J. Fossum E. N. Ingram S. L. Morgan M. M. (2007). Analgesic tolerance to microinjection of the μ-opioid agonist DAMGO into the ventrolateral periaqueductal gray. *Neuropharmacology* 52 1580–1585. 10.1016/J.NEUROPHARM.2007.03.002 17445843 PMC1971241

[B258] Millan M. H. J. Millan M. H. J. Pilcher C. W. T. Cz L. A. Herz A. Colpaert F. C. (1985). Spinal cord dynorphin may modulate nociception via a kappa-opioid receptor in chronic arthritic rats. *Brain Res.* 340 156–159. 10.1016/0006-8993(85)90786-3 2862957

[B259] Millan M. J. Członkowski A. Morris B. Stein C. Arendt R. Huber A. (1988). Inflammation of the hind limb as a model of unilateral, localized pain: influence on multiple opioid systems in the spinal cord of the rat. *Pain* 35 299–312. 10.1016/0304-3959(88)90140-6 2906425

[B260] Millan M. J. Czlonkowski A. Pilcher C. W. T. Almeida O. F. X. Millan M. H. Colpaert F. C. (1987). A model of chronic pain in the rat: functional correlates of alterations in the activity of opioid systems. *J. Neurosci.* 7 77–87. 10.1523/jneurosci.07-01-00077.1987 3027278 PMC6568860

[B261] Minozzi S. Amato L. Davoli M. (2013). Development of dependence following treatment with opioid analgesics for pain relief: a systematic review. *Addiction* 108 688–698. 10.1111/J.1360-0443.2012.04005.X 22775332

[B262] Mitsi V. Zachariou V. (2016). Modulation of pain, nociception, and analgesia by the brain reward center. *Neuroscience* 338 81–92. 27189881 10.1016/j.neuroscience.2016.05.017 PMC5083150

[B263] Mittal N. Roberts K. Pal K. Bentolila L. A. Fultz E. Minasyan A. (2013). Select G-protein-coupled receptors modulate agonist-induced signaling via a ROCK, LIMK, and β-arrestin 1 pathway. *Cell Rep.* 5 1010–1021. 10.1016/J.CELREP.2013.10.015 24239352 PMC3870884

[B264] Miyata H. Takahashi M. Murai Y. Tsuneyoshi K. Hayashi T. Meulien D. (2019). Nalmefene in alcohol-dependent patients with a high drinking risk: randomized controlled trial. *Psychiatry Clin. Neurosci.* 73 697–706. 10.1111/PCN.12914 31298784 PMC6899457

[B265] Mogil J. S. (2012). Pain genetics: past, present and future. *Trends Genet.* 28 258–266. 10.1016/J.TIG.2012.02.004 22464640

[B266] Mogil J. S. Grisel J. E. Reinscheid R. K. Civelli O. Belknap J. K. Grandy D. K. (1996a). Orphanin FQ is a functional anti-opioid peptide. *Neuroscience* 75 333–337. 10.1016/0306-4522(96)00338-7 8930999

[B267] Mogil J. S. Grisel J. E. Zhangs G. Belknap J. K. Grandy D. K. (1996b). Functional antagonism of μ-, δ- and κ-opioid antinociception by orphanin FQ. *Neurosci. Lett.* 214 131–134. 10.1016/0304-3940(96)12917-7 8878101

[B268] Mohammadkhani A. Borgland S. L. (2022). Cellular and behavioral basis of cannabinioid and opioid interactions: implications for opioid dependence and withdrawal. *J. Neurosci. Res.* 100 278–296. 10.1002/jnr.24770 33352618

[B269] Mollereau C. Parmentier M. Mailleux P. Butour J. L. Moisand C. Chalon P. (1994). ORL1, a novel member of the opioid receptor family. Cloning, functional expression and localization. *FEBS Lett.* 341 33–38. 8137918 10.1016/0014-5793(94)80235-1

[B270] Morasco B. J. Gritzner S. Lewis L. Oldham R. Turk D. C. Dobscha S. K. (2011). Systematic review of prevalence, correlates, and treatment outcomes for chronic non-cancer pain in patients with comorbid substance use disorder. *Pain* 152 488–497. 10.1016/J.PAIN.2010.10.009 21185119 PMC3053013

[B271] Morgan D. O. (1997). Cyclin-dependent kinases: engines, clocks, and microprocessors. *Annu. Rev. Cell Dev. Biol.* 13 261–291. 10.1146/ANNUREV.CELLBIO.13.1.261 9442875

[B272] Morgan M. M. Fossum E. N. Levine C. S. Ingram S. L. (2006). Antinociceptive tolerance revealed by cumulative intracranial microinjections of morphine into the periaqueductal gray in the rat. *Pharmacol. Biochem. Behav.* 85 214–219. 10.1016/J.PBB.2006.08.003 16979226

[B273] Morinville A. Cahill C. M. Aibak H. Rymar V. V. Pradhan A. Hoffert C. (2004a). Morphine-induced changes in delta opioid receptor trafficking are linked to somatosensory processing in the rat spinal cord. *J. Neurosci.* 24 5549–5559. 10.1523/JNEUROSCI.2719-03.2004 15201327 PMC6729333

[B274] Morinville A. Cahill C. M. Kieffer B. Collier B. Beaudet A. (2004b). Mu-opioid receptor knockout prevents changes in delta-opioid receptor trafficking induced by chronic inflammatory pain. *Pain* 109 266–273. 10.1016/j.pain.2004.01.011 15157687

[B275] Morone N. E. Weiner D. K. (2013). Pain as the fifth vital sign: exposing the vital need for pain education. *Clin. Ther.* 35 1728–1732. 10.1016/j.clinthera.2013.10.001 24145043 PMC3888154

[B276] Moulédous L. (2019). The nociceptin / orphanin FQ system and the regulation of memory. *Handb. Exp. Pharmacol.* 254 259–278. 30430261 10.1007/164_2018_185

[B277] Moye L. S. Siegersma K. Dripps I. Witkowski W. Mangutov E. Wang D. (2021). Delta opioid receptor regulation of calcitonin gene-related peptide dynamics in the trigeminal complex. *Pain* 162 2297–2308. 10.1097/J.PAIN.0000000000002235 33605657 PMC8730473

[B278] Murakawa K. Hirose N. Takada K. Suzuki T. Nagase H. Cools A. R. (2004). Deltorphin II enhances extracellular levels of dopamine in the nucleus accumbens via opioid receptor-independent mechanisms. *Eur. J. Pharmacol.* 491 31–36. 10.1016/j.ejphar.2004.03.028 15102530

[B279] Murphy N. P. Ly H. T. Maidment N. T. (1996). Intracerebroventricular orphanin FQ/nociceptin suppresses dopamine release in the nucleus accumbens of anaesthetized rats. *Neuroscience* 75 1–4. 10.1016/0306-4522(96)00322-3 8923516

[B280] Nadeau S. E. Wu J. K. Lawhern R. A. (2021). Opioids and chronic pain: an analytic review of the clinical evidence. *Front. Pain Res.* 2:721357. 10.3389/FPAIN.2021.721357 35295493 PMC8915556

[B281] Nakanishi S. Inoue A. Kita T. Inoue A. Nakamura M. Chang A. C. Y. (1979). Nucleotide sequence of cloned cDNA for bovine corticotropin-β- lipotropin precursor. *Nature* 278 423–427. 10.1038/278423a0 221818

[B282] Namba M. D. Leyrer-Jackson J. M. Nagy E. K. Olive M. F. Neisewander J. L. (2021). Neuroimmune mechanisms as novel treatment targets for substance use disorders and associated comorbidities. *Front. Neurosci.* 15:427. 10.3389/FNINS.2021.650785/BIBTEX PMC8082184 33935636

[B283] Narita M. Kaneko C. Miyoshi K. Nagumo Y. Kuzumaki N. Nakajima M. (2006a). Chronic pain induces anxiety with concomitant changes in opioidergic function in the amygdala. *Neuropsychopharmacology* 31 739–750. 10.1038/SJ.NPP.1300858 16123756

[B284] Narita M. Kuzumaki N. Narita M. Kaneko C. Hareyama N. Miyatake M. (2006b). Chronic pain-induced emotional dysfunction is associated with astrogliosis due to cortical δ-opioid receptor dysfunction. *J. Neurochem.* 97 1369–1378. 10.1111/J.1471-4159.2006.03824.X 16696849

[B285] Narita M. Kishimoto Y. Ise Y. Yajima Y. Misawa K. Suzuki T. (2005). Direct evidence for the involvement of the mesolimbic κ-opioid system in the morphine-induced rewarding effect under an inflammatory pain-like state. *Neuropsychopharmacology* 30 111–118. 10.1038/sj.npp.1300527 15257306

[B286] Navratilova E. Atcherley C. W. Porreca F. (2015). Brain circuits encoding reward from pain relief. *Trends Neurosci.* 38 741–750. 10.1016/J.TINS.2015.09.003 26603560 PMC4752429

[B287] Navratilova E. Ji G. Phelps C. Qu C. Hein M. Yakhnitsa V. (2019). Kappa opioid signaling in the central nucleus of the amygdala promotes disinhibition and aversiveness of chronic neuropathic pain. *Pain* 160 824–832. 10.1097/J.PAIN.0000000000001458 30681985 PMC6424634

[B288] Nguyen D. Alushaj E. Erb S. Ito R. (2019). Dissociative effects of dorsomedial striatum D1 and D2 receptor antagonism in the regulation of anxiety and learned approach-avoidance conflict decision-making. *Neuropharmacology* 146 222–230. 10.1016/j.neuropharm.2018.11.040 30508508

[B289] NIDA (2008). *Comorbidity: Addiction and Other Mental Illnesses.* Bethesda, MD: NIDA.

[B290] Niikura K. Narita M. Butelman E. R. Kreek M. J. Suzuki T. (2010). Neuropathic and chronic pain stimuli downregulate central μ -opioid and dopaminergic transmission. *Trends Pharmacol. Sci.* 31 299–305. 10.1016/J.TIPS.2010.04.003 20471111

[B291] Niikura K. Narita M. Narita M. Nakamura A. Okutsu D. Ozeki A. (2008). Direct evidence for the involvement of endogenous β-endorphin in the suppression of the morphine-induced rewarding effect under a neuropathic pain-like state. *Neurosci. Lett.* 435 257–262. 10.1016/J.NEULET.2008.02.059 18359165

[B292] Normandin A. Luccarini P. Molat J. L. Gendron L. Dallel R. (2013). Spinal μ and δ opioids inhibit both thermal and mechanical pain in rats. *J. Neurosci.* 33 11703–11714. 10.1523/JNEUROSCI.1631-13.2013 23843537 PMC3855450

[B293] Nylander I. Vlaskovska M. Terenius L. (1995). Brain dynorphin and enkephalin systems in Fischer and Lewis rats: effects of morphine tolerance and withdrawal. *Brain Res.* 683 25–35. 10.1016/0006-8993(95)00279-Y 7552341

[B294] Olson G. A. Olson R. D. Kastin A. J. Coy D. H. (1979). Endogenous opiates: through 1978. *Neurosci. Biobehav. Rev.* 3 285–299. 10.1016/0149-7634(79)90014-9 232257

[B295] Ozaki S. Narita M. Narita M. Iino M. Miyoshi K. Suzuki T. (2003). Suppression of the morphine-induced rewarding effect and G-protein activation in the lower midbrain following nerve injury in the mouse: involvement of G-protein-coupled receptor kinase 2. *Neuroscience* 116 89–97. 10.1016/S0306-4522(02)00699-1 12535942

[B296] Ozaki S. Narita M. Narita M. Ozaki M. Khotib J. Suzuki T. (2004). Role of extracellular signal-regulated kinase in the ventral tegmental area in the suppression of the morphine-induced rewarding effect in mice with sciatic nerve ligation. *J. Neurochem.* 88 1389–1397. 10.1046/J.1471-4159.2003.02272.X 15009639

[B297] Ozaki S. Narita M. M. Narita M. M. Iino M. Sugita J. Matsumura Y. (2002). Suppression of the morphine-induced rewarding effect in the rat with neuropathic pain: implication of the reduction in l-opioid receptor functions in the ventral tegmental area. *J. Neurochem.* 82 1192–1198. 10.1046/J.1471-4159.2002.01071.X 12358766

[B298] Parida S. Carroll K. M. Petrakis I. L. Sofuoglu M. (2019). Buprenorphine treatment for opioid use disorder: recent progress. *Expert Rev. Clin. Pharmacol.* 12 791–803. 10.1080/17512433.2019.1635454 31232604

[B299] Pathan H. Williams J. (2012). Basic opioid pharmacology: an update. *Br. J. Pain* 6:11. 10.1177/2049463712438493 26516461 PMC4590096

[B300] Peciña M. Karp J. F. Mathew S. Todtenkopf M. S. Ehrich E. W. Zubieta J. K. (2018). Endogenous opioid system dysregulation in depression: implications for new therapeutic approaches. *Mol. Psychiatry* 24 576–587. 10.1038/s41380-018-0117-2 29955162 PMC6310672

[B301] Peng J. Sarkar S. Chang S. L. (2012). Opioid receptor expression in human brain and peripheral tissues using absolute quantitative real-time RT-PCR. *Drug Alcohol. Depend.* 124 223–228. 10.1016/J.DRUGALCDEP.2012.01.013 22356890 PMC3366045

[B302] Pereira F. G. França M. H. de Paiva M. C. A. Andrade L. H. Viana M. C. (2017). Prevalence and clinical profile of chronic pain and its association with mental disorders. *Rev. Saude Publ.* 51:96. 10.11606/S1518-8787.2017051007025 29166447 PMC5676726

[B303] Pergolizzi J. Jr. Raffa R. (2019). Safety and efficacy of the unique opioid buprenorphine for the treatment of chronic pain. *J. Pain Res.* 12 3299–3317. 10.2147/JPR.S231948 31997882 PMC6917545

[B304] Perrine S. A. Hoshaw B. A. Unterwald E. M. (2006). Delta opioid receptor ligands modulate anxiety-like behaviors in the rat. *Br. J. Pharmacol.* 147 864–872. 10.1038/SJ.BJP.0706686 16491101 PMC1760715

[B305] Pert C. B. Snyder S. H. (1973). Opiate receptor: demonstration in nervous tissue. *Science* 179 1011–1014. 10.1126/SCIENCE.179.4077.1011 4687585

[B306] Petraschka M. Li S. Gilbert T. L. Westenbroek R. E. Bruchas M. R. Schreiber S. (2007). The absence of endogenous beta-endorphin selectively blocks phosphorylation and desensitization of mu opioid receptors following partial sciatic nerve ligation. *Neuroscience* 146 1795–1807. 10.1016/J.NEUROSCIENCE.2007.03.029 17467916 PMC2012364

[B307] Pfeiffer A. Brantl V. Herz A. Emrich H. M. (1986). Psychotomimesis mediated by kappa opiate receptors. *Science* 233 774–776. 10.1126/SCIENCE.3016896 3016896

[B308] Pignatelli M. Bonci A. (2015). Role of dopamine neurons in reward and aversion: a synaptic plasticity perspective. *Neuron* 86 1145–1157. 10.1016/j.neuron.2015.04.015 26050034

[B309] Pisanu C. Franconi F. Gessa G. L. Mameli S. Pisanu G. M. Campesi I. (2019). Sex differences in the response to opioids for pain relief: a systematic review and meta-analysis. *Pharmacol. Res.* 148:104447. 10.1016/J.PHRS.2019.104447 31499196 PMC12522423

[B310] Podvin S. Yaksh T. Hook V. (2016). The emerging role of spinal dynorphin in chronic pain: a therapeutic perspective. *Annu. Rev. Pharmacol. Toxicol.* 56 511–533. 10.1146/annurev-pharmtox-010715-103042 26738478 PMC4902163

[B311] Pol O. Murtra P. Caracuel L. Valverde O. Puig M. M. Maldonado R. (2006). Expression of opioid receptors and c-fos in CB1 knockout mice exposed to neuropathic pain. *Neuropharmacology* 50 123–132. 10.1016/J.NEUROPHARM.2005.11.002 16360182

[B312] Porreca F. Tang Q. B. Bian D. Riedl M. Eide R. Lai J. (1998). Spinal opioid mu receptor expression in lumbar spinal cord of rats following nerve injury. *Brain Res.* 795 197–203. 10.1016/S0006-8993(98)00292-3 9622629

[B313] Pradhan A. A. Befort K. Nozaki C. Gavériaux-Ruff C. Kieffer B. L. (2011). The delta opioid receptor: an evolving target for the treatment of brain disorders. *Trends Pharmacol. Sci.* 32 581–590. 10.1016/J.TIPS.2011.06.008 21925742 PMC3197801

[B314] Przewlocki R. (2004). Opioid abuse and brain gene expression. *Eur. J. Pharmacol.* 500 331–349. 10.1016/J.EJPHAR.2004.07.036 15464044

[B315] Quirion B. Bergeron F. Blais V. Gendron L. (2020). The delta-opioid receptor; a target for the treatment of pain. *Front. Mol. Neurosci.* 13:52. 10.3389/fnmol.2020.00052 32431594 PMC7214757

[B316] Raehal K. M. Schmid C. L. Groer C. E. Bohn L. M. (2011). Functional selectivity at the μ-opioid receptor: implications for understanding opioid analgesia and tolerance. *Pharmacol. Rev.* 63 1001–1019. 10.1124/pr.111.004598 21873412 PMC3186080

[B317] Raffa R. B. Martinez R. P. Connelly C. D. (1994). G-protein antisense oligodeoxyribonucleotides and μ-opioid supraspinal antinociception. *Eur. J. Pharmacol.* 258 R5–R7. 10.1016/0014-2999(94)90073-6 7925586

[B318] Raghavendra V. Rutkowski M. D. Deleo J. A. (2002). The role of spinal neuroimmune activation in morphine tolerance/hyperalgesia in neuropathic and sham-operated rats. *J. Neurosci.* 22 9980–9989. 10.1523/JNEUROSCI.22-22-09980.2002 12427855 PMC6757841

[B319] Raja S. N. Carr D. B. Cohen M. Finnerup N. B. Flor H. Gibson S. (2020). The Revised IASP definition of pain: concepts, challenges, and compromises. *Pain* 161:1976. 10.1097/J.PAIN.0000000000001939 32694387 PMC7680716

[B320] Ranganathan M. Schnakenberg A. Skosnik P. D. Cohen B. M. Pittman B. Sewell R. A. (2012). Dose-related behavioral, subjective, endocrine, and psychophysiological effects of the κ opioid agonist Salvinorin A in humans. *Biol. Psychiatry* 72 871–879. 10.1016/J.BIOPSYCH.2012.06.012 22817868 PMC3638802

[B321] Rashid H. Inoue M. Toda K. Ueda H. (2004). Loss of peripheral morphine analgesia contributes to the reduced effectiveness of systemic morphine in neuropathic pain. *J. Pharmacol. Exp. Ther.* 309 380–387. 10.1124/JPET.103.060582 14718584

[B322] Reiner D. J. Fredriksson I. Lofaro O. M. Bossert J. M. Shaham Y. (2019). Relapse to opioid seeking in rat models: behavior, pharmacology and circuits. *Neuropsychopharmacology* 44 465–477. 10.1038/s41386-018-0234-2 30293087 PMC6333846

[B323] Reiner D. J. Townsend E. A. Orihuel J. Applebey S. V. Claypool S. M. Banks M. L. (2021). Lack of effect of different pain-related manipulations on opioid self-administration, reinstatement of opioid seeking, and opioid choice in rats. *Psychopharmacology* 238 1885–1897. 10.1007/S00213-021-05816-9/FIGURES/5 33765177 PMC10041878

[B324] Reinscheid R. K. Nothacker H. P. Bourson A. Ardati A. Henningsen R. A. Bunzow J. R. (1995). Orphanin FQ: a neuropeptide that activates an opioidlike G protein-coupled receptor. *Science* 270 792–794. 7481766 10.1126/science.270.5237.792

[B325] Reiss D. Maurin H. Audouard E. Martínez-Navarro M. Xue Y. Herault Y. (2021). Delta opioid receptor in astrocytes contributes to neuropathic cold pain and analgesic tolerance in female mice. *Front. Cell Neurosci.* 15:745178. 10.3389/FNCEL.2021.745178/FULL 34602984 PMC8483180

[B326] Remesic M. Hruby V. J. Porreca F. Lee Y. S. (2017). Recent advances in the realm of allosteric modulators for opioid receptors for future therapeutics. *ACS Chem. Neurosci.* 8 1147–1158. 10.1021/ACSCHEMNEURO.7B00090 28368571 PMC5689070

[B327] Ren W. Centeno M. V. Berger S. Wu Y. Na X. Liu X. (2015). The indirect pathway of the nucleus accumbens shell amplifies neuropathic pain. *Nat. Neurosci.* 19 220–222. 10.1038/nn.4199 26691834 PMC4889808

[B328] Ren W. Centeno M. V. Wei X. Wickersham I. Martina M. Apkarian A. V. (2021). Adaptive alterations in the mesoaccumbal network after peripheral nerve injury. *Pain* 162 895–906. 10.1097/J.PAIN.0000000000002092 33021562 PMC9272541

[B329] Rhodin A. Grönbladh A. Ginya H. Nilsson K. W. Rosenblad A. Zhou Q. (2013). Combined analysis of circulating β-endorphin with gene polymorphisms in OPRM1, CACNAD2 and ABCB1 reveals correlation with pain, opioid sensitivity and opioid-related side effects. *Mol. Brain* 6:8. 10.1186/1756-6606-6-8 23402298 PMC3602034

[B330] Ringkamp M. Dougherty P. M. Raja S. N. (2018). Anatomy and physiology of the pain signaling process. *Essent. Pain Med.* 2018 3.e1–10.e1. 10.1016/B978-0-323-40196-8.00001-2

[B331] Rizvi S. J. Gandhi W. Salomons T. (2021). Reward processing as a common diathesis for chronic pain and depression. *Neurosci. Biobehav. Rev.* 127 749–760. 10.1016/J.NEUBIOREV.2021.04.033 33951413

[B332] Rizzi A. Bigoni R. Marzola G. Guerrini R. Salvadori S. Regoli D. (2001). Characterization of the locomotor activity-inhibiting effect of nociceptin/orphanin FQ in mice. *Naunyn Schmiedebergs Arch. Pharmacol.* 363 161–165. 10.1007/s002100000358 11218068

[B333] Rizzi A. Gavioli E. Marzola G. Spagnolo B. Zucchini S. Ciccocioppo R. (2007). Pharmacological characterization of the nociceptin/orphanin FQ receptor antagonist SB-612111 [(–)-cis-1-Methyl-7-[[4-(2,6-dichlorophenyl)piperidin-1-yl]methyl]-6,7,8,9-tetrahydro-5H-benzocyclohepten-5-ol]: *in vivo* studies. *J. Pharmacol. Exp. Ther.* 321 968–974. 17329551 10.1124/jpet.106.116780

[B334] Rizzi A. Molinari S. Marti M. Marzola G. Calo’ G. (2011). Nociceptin/orphanin FQ receptor knockout rats: *in vitro* and *in vivo* studies. *Neuropharmacology* 60 572–579. 10.1016/J.NEUROPHARM.2010.12.010 21184763

[B335] Roeckel L. A. le Coz G. M. Gavériaux-Ruff C. Simonin F. (2016). Opioid-induced hyperalgesia: cellular and molecular mechanisms. *Neuroscience* 338 160–182. 10.1016/j.neuroscience.2016.06.029 27346146

[B336] Rogers A. H. Manning K. Garey L. Smit T. Zvolensky M. J. (2020). Sex differences in the relationship between anxiety sensitivity and opioid misuse among adults with chronic pain. *Addict. Behav.* 102:106156. 10.1016/J.ADDBEH.2019.106156 31704430

[B337] Rudd R. A. Aleshire N. Zibbell J. E. Gladden R. M. (2016). Increases in drug and opioid overdose deaths—United States, 2000–14. *Morb. Mortal Wkly Rep.* 64 1378–1382. 10.15585/mmwr.mm6450a3 26720857

[B338] Saegusa H. Kurihara T. Zong S. Minowa O. Kazuno A.-A. Han W. (2000). *Altered Pain Responses in Mice Lacking 1E Subunit of the Voltage-Dependent Ca 2 Channel.* Stanford, CA: Stanford University School of Medicine. 10.1073/pnas.100124197 PMC18570 10801976

[B339] Saigusa T. Aono Y. Waddington J. L. (2017). Mechanisms underlying δ- and μ-opioid receptor agonist-induced increases in extracellular dopamine level in the nucleus accumbens of freely moving rats. *J. Oral Sci.* 59 195–200. 10.2334/josnusd.16-0874 28637978

[B340] Saitoh A. Yoshikawa Y. Onodera K. Kamei J. (2005). Role of δ-opioid receptor subtypes in anxiety-related behaviors in the elevated plus-maze in rats. *Psychopharmacology* 182 327–334. 10.1007/S00213-005-0112-6 16075288

[B341] Sakoori K. Murphy N. P. (2004). Central administration of nociceptin/orphanin FQ blocks the acquisition of conditioned place preference to morphine and cocaine, but not conditioned place aversion to naloxone in mice. *Psychopharmacology* 172 129–136. 10.1007/S00213-003-1643-3 14624329

[B342] Samuelsonn H. Ekman R. Hedner T. (1993). CSF neuropeptides in cancer pain: effects of spinal opioid therapy. *Acta Anaesthesiol. Scand.* 37 502–508. 10.1111/J.1399-6576.1993.TB03755.X 8356865

[B343] Schlosburg J. E. Whitfield T. W. Park P. E. Crawford E. F. George O. Vendruscolo L. F. (2013). Long-term antagonism of κ opioid receptors prevents escalation of and increased motivation for heroin intake. *J. Neurosci.* 33 19384–19392. 10.1523/JNEUROSCI.1979-13.2013 24305833 PMC3850049

[B344] Schrepf A. Harper D. E. Harte S. E. Wang H. Ichesco E. Hampson J. P. (2016). Endogenous opioidergic dysregulation of pain in fibromyalgia: a PET and fMRI study. *Pain* 157 2217–2225. 10.1097/J.PAIN.0000000000000633 27420606 PMC5028286

[B345] Schwartz N. Temkin P. Jurado S. Lim B. K. Heifets B. D. Polepalli J. S. (2014). Decreased motivation during chronic pain requires long-term depression in the nucleus accumbens. *Science* 345 535–542. 10.1126/science.1253994 25082697 PMC4219555

[B346] Scoto G. M. Aricò G. Iemolo A. Ronsisvalle G. Parenti C. (2010). Selective inhibition of the NOP receptor in the ventrolateral periaqueductal gray attenuates the development and the expression of tolerance to morphine-induced antinociception in rats. *Peptides* 31 696–700. 10.1016/j.peptides.2009.12.028 20067813

[B347] Selley D. E. Lazenka M. F. Sim-Selley L. J. Secor McVoy J. R. Potter D. N. Chartoff E. H. (2020). Attenuated dopamine receptor signaling in nucleus accumbens core in a rat model of chemically-induced neuropathy. *Neuropharmacology* 166:107935. 10.1016/J.NEUROPHARM.2020.107935 31917153 PMC7654441

[B348] Shen C. H. Tsai R. Y. Shih M. S. Lin S. L. Tai Y. H. Chien C. C. (2011). Etanercept restores the antinociceptive effect of morphine and suppresses spinal neuroinflammation in morphine-tolerant rats. *Anesth. Analg.* 112 454–459. 10.1213/ANE.0B013E3182025B15 21081778

[B349] Shenoy S. S. Lui F. (2018). *Biochemistry, Endogenous Opioids.* Treasure Island, FL: StatPearls Publishing. 30422494

[B350] Shi J. Zhao L. Y. Copersino M. L. Fang Y. X. Chen Y. Tian J. (2008). PET imaging of dopamine transporter and drug craving during methadone maintenance treatment and after prolonged abstinence in heroin users. *Eur. J. Pharmacol.* 579 160–166. 10.1016/J.EJPHAR.2007.09.042 17977528

[B351] Shimoyama N. Shimoyama M. Elliott K. J. Inturrisi C. E. (1997). d-methadone is antinociceptive in the rat formalin test. *J. Pharmacol. Exp. Ther.* 283 648–652. 9353381

[B352] Shoblock J. R. Wichmann J. Maidment N. T. (2005). The effect of a systemically active ORL-1 agonist, Ro 64-6198, on the acquisition, expression, extinction, and reinstatement of morphine conditioned place preference. *Neuropharmacology* 49 439–446. 10.1016/J.NEUROPHARM.2005.04.008 15919100

[B353] Shulman M. Luo S. X. Campbell A. N. C. Scodes J. Pavlicova M. Broffman A. (2020). Secondary analysis of pain outcomes in a large pragmatic randomized trial of buprenorphine/naloxone versus methadone for opioid use disorder. *J. Addict. Med.* 14 e188–e194. 10.1097/ADM.0000000000000630 32039934 PMC7415472

[B354] Silverman S. M. (2009). Opioid induced hyperalgesia: clinical implications for the pain practitioner. *Pain Phys.* 12 679–684. 10.36076/ppj.2009/12/679 19461836

[B355] Simon E. J. Hiller J. M. Edelman I. (1973). Stereospecific binding of the potent narcotic analgesic [3H] etorphine to rat brain homogenate (opiate receptor/morphine/antagonist). *Proc. Natl. Acad. Sci. U.S.A.* 70 1947–1949. 10.1073/pnas.70.7.1947 4516196 PMC433639

[B356] Siuda E. R. Carr R. Rominger D. H. Violin J. D. (2017). Biased mu-opioid receptor ligands: a promising new generation of pain therapeutics. *Curr. Opin. Pharmacol.* 32 77–84. 10.1016/j.coph.2016.11.007 27936408

[B357] Sjøgren P. Jensen N. H. Jensen T. S. (1994). Disappearance of morphine-induced hyperalgesia after discontinuing or substituting morphine with other opioid agonists. *Pain* 59 313–316. 10.1016/0304-3959(94)90084-1 7892029

[B358] Sliepen S. H. J. Korioth J. Christoph T. Tzschentke T. M. Diaz-delCastillo M. Heegaard A. M. (2021). The nociceptin/orphanin FQ receptor system as a target to alleviate cancer-induced bone pain in rats: model validation and pharmacological evaluation. *Br. J. Pharmacol.* 178 1995–2007. 10.1111/BPH.14899 31724155 PMC8246843

[B359] Smit T. Rogers A. H. Garey L. Allan N. P. Viana A. G. Zvolensky M. J. (2020). Anxiety sensitivity and pain intensity independently predict opioid misuse and dependence in chronic pain patients. *Psychiatry Res.* 294:113523. 10.1016/J.PSYCHRES.2020.113523 33189986

[B360] Soares F. H. C. Kubota G. T. Fernandes A. M. Hojo B. Couras C. Costa B. V. (2021). Prevalence and characteristics of new-onset pain in COVID-19 survivours, a controlled study. *Eur. J. Pain* 25 1342–1354. 10.1002/EJP.1755 33619793 PMC8013219

[B361] Solecki W. Ziolkowska B. Krowka T. Gieryk A. Filip M. Przewlocki R. (2009). Alterations of prodynorphin gene expression in the rat mesocorticolimbic system during heroin self-administration. *Brain Res.* 1255 113–121. 10.1016/J.BRAINRES.2008.12.002 19100723

[B362] Spahn V. del Vecchio G. Labuz D. Rodriguez-Gaztelumendi A. Massaly N. Temp J. (2017). A nontoxic pain killer designed by modeling of pathological receptor conformations. *Science* 355 966–969. 10.1126/SCIENCE.AAI8636 28254944

[B363] Spanagel R. Herz A. Shippenberg T. S. (1992). Opposing tonically active endogenous opioid systems modulate the mesolimbic dopaminergic pathway. *Proc. Natl. Acad. Sci. U.S.A.* 89 2046–2050. 10.1073/PNAS.89.6.2046 1347943 PMC48593

[B364] Spetea M. van Rijn R. M. Daibani A. Che T. (2022). Spotlight on nociceptin/orphanin FQ receptor in the treatment of pain. *Molecules* 27:595. 10.3390/MOLECULES27030595 35163856 PMC8838650

[B365] Stein B. D. Sherry T. B. O’Neill B. Taylor E. A. Sorbero M. (2021). Rapid discontinuation of chronic, high-dose opioid treatment for pain: prevalence and associated factors. *J. Gen. Intern. Med.* 37 1603–1609. 10.1007/S11606-021-07119-3/TABLES/2 34608565 PMC9130349

[B366] Stein C. (2013). Opioids, sensory systems and chronic pain. *Eur. J. Pharmacol.* 716 179–187. 10.1016/J.EJPHAR.2013.01.076 23500206

[B367] Stein C. (2016). Opioid receptors. *Annu. Rev. Med.* 67 433–451. 10.1146/ANNUREV-MED-062613-093100 26332001

[B368] Stein C. Clark J. D. Oh U. Vasko M. R. Wilcox G. L. Overland A. C. (2009). Peripheral mechanisms of pain and analgesia. *Brain Res. Rev.* 60 90–113. 10.1016/j.brainresrev.2008.12.017 19150465 PMC2730351

[B369] Stein C. Schäfer M. Machelska H. (2003). Attacking pain at its source: new perspectives on opioids. *Nat. Med.* 9 1003–1008. 10.1038/nm908 12894165

[B370] Stevenson G. W. Folk J. E. Rice K. C. Negus S. S. (2005). Interactions between δ and μ opioid agonists in assays of schedule-controlled responding, thermal nociception, drug self-administration, and drug versus food choice in rhesus monkeys: studies with SNC80 [(+)-4-[(αR)-α-((2S,5R)-4-allyl-2,5-dimethyl-1-piperazinyl) -3-methoxybenzyl]-N,N-diethylbenzamide] and heroin. *J. Pharmacol. Exp. Ther.* 314 221–231. 10.1124/jpet.104.082685 15792997

[B371] Stoeber M. Jullié D. Lobingier B. T. Laeremans T. Steyaert J. Schiller P. W. (2018). A genetically encoded biosensor reveals location bias of opioid drug action. *Neuron* 98 963.e5–976.e5. 10.1016/j.neuron.2018.04.021 29754753 PMC6481295

[B372] Sturgeon J. A. Sullivan M. D. Parker-Shames S. Tauben D. Coelho P. (2020). Outcomes in long-term opioid tapering and buprenorphine transition: a retrospective clinical data analysis. *Pain Med.* 21 3635–3644. 10.1093/pm/pnaa029 32163149

[B373] Taylor A. M. W. Castonguay A. Taylor A. J. Murphy N. P. Ghogha A. Cook C. (2015). Microglia disrupt mesolimbic reward circuitry in chronic pain. *J. Neurosci.* 35:8442. 10.1523/JNEUROSCI.4036-14.2015 26041913 PMC4452552

[B374] Taylor A. M. W. W. Becker S. Schweinhardt P. Cahill C. (2016). Mesolimbic dopamine signaling in acute and chronic pain: implications for motivation, analgesia, and addiction. *Pain* 157:1194. 10.1097/j.pain.0000000000000494 26797678 PMC4866581

[B375] Tejeda H. A. Bonci A. (2019). Dynorphin/kappa-opioid receptor control of dopamine dynamics: implications for negative affective states and psychiatric disorders. *Brain Res.* 1713 91–101. 10.1016/J.BRAINRES.2018.09.023 30244022

[B376] Tejeda H. A. Counotte D. S. Oh E. Ramamoorthy S. Chefer V. Donnell P. O. (2013). Prefrontal cortical kappa-opioid receptor modulation of local neurotransmission and conditioned place aversion. *Neuropsychopharmacology* 38 1770–1779. 10.1038/npp.2013.76 23542927 PMC3717537

[B377] Tejeda H. A. Wu J. Kornspun A. R. Pignatelli M. Kashtelyan V. Krashes M. J. (2017). Pathway- and cell-specific kappa-opioid receptor modulation of excitation-inhibition balance differentially gates D1 and D2 accumbens neuron activity. *Neuron* 93 147–163. 10.1016/j.neuron.2016.12.005 28056342 PMC5808882

[B378] Terenius L. (1973). Stereospecific interaction between narcotic analgesics and a synaptic plasma membrane fraction of rat cerebral cortex. *Acta Pharmacol. Toxicol.* 32 317–320. 10.1111/j.1600-0773.1973.tb01477.x 4801733

[B379] Thompson S. J. Pitcher M. H. Stone L. S. Tarum F. Niu G. Chen X. (2018). Chronic neuropathic pain reduces opioid receptor availability with associated anhedonia in rat. *Pain* 159 1856–1866. 10.1097/J.PAIN.0000000000001282 29794614 PMC6095806

[B380] Toll L. Bruchas M. R. Calo’ G. Cox B. M. Zaveri N. T. (2016). Nociceptin/Orphanin FQ receptor structure, signaling, ligands, functions, and interactions with opioid systems. *Pharmacol. Rev.* 68 419–457. 10.1124/PR.114.009209 26956246 PMC4813427

[B381] Torregrossa M. M. Jutkiewicz E. M. Mosberg H. I. Balboni G. Watson S. J. Woods J. H. (2006). Peptidic delta opioid receptor agonists produce antidepressant-like effects in the forced swim test and regulate BDNF mRNA expression in rats. *Brain Res.* 1069 172–181. 10.1016/j.brainres.2005.11.005 16364263 PMC1780167

[B382] Toubia T. Khalife T. (2019). The endogenous opioid system: role and dysfunction caused by opioid therapy. *Clin. Obstet. Gynecol.* 62 3–10. 10.1097/GRF.0000000000000409 30398979

[B383] Tremblay J. Hamet P. (2010). Genetics of pain, opioids, and opioid responsiveness. *Metabolism* 59 S5–S8. 10.1016/J.METABOL.2010.07.015 20837195

[B384] Trujillo K. A. Bronstein D. M. Sanchez I. O. Akil H. (1995). Effects of chronic opiate and opioid antagonist treatment on striatal opioid peptides. *Brain Res.* 698 69–78. 10.1016/0006-8993(95)00809-5 8581505

[B385] Truong W. Cheng C. Xu Q. G. Li X. Q. Zochodne D. W. (2003). μ Opioid receptors and analgesia at the site of a peripheral nerve injury. *Ann. Neurol.* 53 366–375. 10.1002/ana.10465 12601704

[B386] Tzschentke T. M. Linz K. Frosch S. Christoph T. (2017). Antihyperalgesic, antiallodynic, and antinociceptive effects of cebranopadol, a novel potent nociceptin/orphanin FQ and opioid receptor agonist, after peripheral and central administration in rodent models of neuropathic pain. *Pain Pract.* 17 1032–1041. 10.1111/papr.12558 28112482

[B387] Tzschentke T. M. Linz K. Koch T. Christoph T. (2019). Cebranopadol: a novel first-in-class potent analgesic acting via NOP and opioid receptors. *Handb. Exp. Pharmacol.* 254 367–398. 10.1007/164_2019_206 30927089

[B388] U.S. Department of Health and Human Services (2019). *Pain Management Best Practices Inter-Agency Task Force Report: Updates, Gaps, Inconsistencies, and Recommendations.* Washington, DC: U.S. Department of Health and Human Services.

[B389] Ueda H. Yamaguchi T. Tokuyama S. Inoue M. Nishi M. Takeshima H. (1997). Partial loss of tolerance liability to morphine analgesia in mice lacking the nociceptin receptor gene. *Neurosci. Lett.* 237 136–138. 10.1016/S0304-3940(97)00832-X 9453234

[B390] Ur E. Wright D. M. Bouloux P. M. G. Grossman A. (1997). The effects of spiradoline (U-62066E), a kappa-opioid receptor agonist, on neuroendocrine function in man. *Br. J. Pharmacol.* 120 781–784. 10.1038/SJ.BJP.0700971 9138682 PMC1564535

[B391] Valentino R. J. Volkow N. D. (2018). Untangling the complexity of opioid receptor function. *Neuropsychopharmacology* 43 2514–2520. 10.1038/s41386-018-0225-3 30250308 PMC6224460

[B392] Vanderah T. W. Suenaga N. M. H. Ossipov M. H. Malan J. Lai J. Porreca F. (2001). Tonic descending facilitation from the rostral ventromedial medulla mediates opioid-induced abnormal pain and antinociceptive tolerance. *J. Neurosci.* 21 279–286. 10.1523/jneurosci.21-01-00279.2001 11150345 PMC6762454

[B393] Vergara F. Sardi N. F. Pescador A. C. Guaita G. O. Jark Stern C. A. Chichorro J. G. (2020). Contribution of mesolimbic dopamine and kappa opioid systems to the transition from acute to chronic pain. *Neuropharmacology* 178:108226. 10.1016/J.NEUROPHARM.2020.108226 32771527

[B394] Viana M. C. Lim C. C. W. Garcia Pereira F. Aguilar-Gaxiola S. Alonso J. Bruffaerts R. (2018). Previous mental disorders and subsequent onset of chronic back or neck pain: findings from 19 countries. *J. Pain* 19 99–110. 10.1016/J.JPAIN.2017.08.011 29031785 PMC6839827

[B395] Volkow N. Benveniste H. McLellan A. T. (2018). Use and misuse of opioids in chronic pain. *Annu. Rev. Med.* 69 451–465. 10.1146/ANNUREV-MED-011817-044739 29029586

[B396] Volkow N. D. McLellan A. T. (2016). Opioid abuse in chronic pain — misconceptions and mitigation strategies. *New Engl. J. Med.* 374 1253–1263. 10.1056/NEJMRA1507771 27028915

[B397] Vollstädt-Klein S. Bumb J. M. Otto A. Dinter C. Karl D. Koopmann A. (2019). The effects of nalmefene on emotion processing in alcohol use disorder - A randomized, controlled fMRI study. *Eur. Neuropsychopharmacol.* 29 1442–1452. 10.1016/J.EURONEURO.2019.10.014 31740271

[B398] Voon P. Karamouzian M. Kerr T. (2017). Chronic pain and opioid misuse: a review of reviews. *Subst. Abuse Treat. Prev. Policy* 12 1–9. 10.1186/S13011-017-0120-7/TABLES/3 28810899 PMC5558770

[B399] Vowles K. E. McEntee M. L. Julnes P. S. Frohe T. Ney J. P. van der Goes D. N. (2015). Rates of opioid misuse, abuse, and addiction in chronic pain: a systematic review and data synthesis. *Pain* 156 569–576. 10.1097/01.j.pain.0000460357.01998.f1 25785523

[B400] Wade C. L. Krumenacher P. Kitto K. F. Peterson C. D. Wilcox G. L. Fairbanks C. A. (2013). Effect of chronic pain on fentanyl self-administration in mice. *PLoS One* 8:e79239. 10.1371/journal.pone.0079239 24260176 PMC3829846

[B401] Wakaizumi K. Jabakhanji R. Ihara N. Kosugi S. Terasawa Y. Morisaki H. (2019). Altered functional connectivity associated with time discounting in chronic pain. *Sci. Rep.* 9:8154. 10.1038/S41598-019-44497-5 31148557 PMC6544657

[B402] Walker J. R. Spina M. Terenius L. Koob G. F. (1998). Nociceptin fails to affect heroin self-administration in the rat. *Neuroreport* 9 2243–2247. 10.1097/00001756-199807130-00017 9694207

[B403] Walsh S. L. Preston K. L. Stitzer M. L. Cone E. J. Bigelow G. E. (1994). Clinical pharmacology of buprenorphine: ceiling effects at high doses. *Clin. Pharmacol. Ther.* 55 569–580. 10.1038/clpt.1994.71 8181201

[B404] Wandner L. D. Domenichiello A. F. Beierlein J. Pogorzala L. Aquino G. Siddons A. (2022). NIH’s helping to end addiction long-termSM initiative (NIH HEAL initiative) clinical pain management common data element program. *J. Pain* 23 370–378. 10.1016/J.JPAIN.2021.08.005 34508905

[B405] Wang D. Tawfik V. L. Corder G. Low S. A. François A. Basbaum A. I. (2018). Functional divergence of Delta and Mu opioid receptor organization in CNS pain circuits. *Neuron* 98 90.e5–108.e5. 10.1016/J.NEURON.2018.03.002 29576387 PMC5896237

[B406] Wang H. Zhang Y. Ma X. Wang W. Xu X. Huang M. (2020). Spinal TLR4/P2X7 receptor-dependent NLRP3 inflammasome activation contributes to the development of tolerance to morphine-induced antinociception. *J. Inflamm. Res.* 13 571–582. 10.2147/JIR.S266995 33061523 PMC7522404

[B407] Wang H. Y. Friedman E. Olmstead M. C. Burns L. H. (2005). Ultra-low-dose naloxone suppresses opioid tolerance, dependence and associated changes in mu opioid receptor–G protein coupling and Gβγ signaling. *Neuroscience* 135 247–261. 10.1016/J.NEUROSCIENCE.2005.06.003 16084657

[B408] Ward J. Hall W. Mattick R. (2009). “Methadone maintenance treatment,” in *Pharmacotherapies for the Treatment of Opioid Dependence: Efficacy, Cost-Effectiveness, and Implementation Guidelines*, eds Mattick R. P. Ali R. Lintzeris N. (Boca Raton, FL: CRC Press), 107–141.

[B409] Wardlaw S. L. Kim J. Sobieszczyk S. (1996). Effect of morphine on proopiomelanocortin gene expression and peptide levels in the hypothalamus. *Brain Res. Mol. Brain Res.* 41 140–147. 10.1016/0169-328X(96)00084-8 8883945

[B410] Wawrzczak-Bargieła A. Ziółkowska B. Piotrowska A. Starnowska-Sokół J. Rojewska E. Mika J. (2020). Neuropathic pain dysregulates gene expression of the forebrain opioid and dopamine systems. *Neurotox Res.* 37 800–814. 10.1007/S12640-020-00166-4 32026358 PMC7085470

[B411] Williams J. T. Christie M. J. Manzoni O. (2001). Cellular and synaptic adaptations mediating opioid dependence. *Physiol. Rev.* 81 299–343. 10.1152/PHYSREV.2001.81.1.299 11152760

[B412] Wilsey B. L. Fishman S. M. Tsodikov A. Ogden C. Symreng I. Ernst A. (2008). Psychological comorbidities predicting prescription opioid abuse among patients in chronic pain presenting to the emergency department. *Pain Med.* 9 1107–1117. 10.1111/J.1526-4637.2007.00401.X 18266809

[B413] Witkin J. M. Statnick M. A. Rorick-Kehn L. M. Pintar J. E. Ansonoff M. Chen Y. (2014). The biology of Nociceptin/Orphanin FQ (N/OFQ) related to obesity, stress, anxiety, mood, and drug dependence. *Pharmacol. Ther.* 141 283–299. 10.1016/J.PHARMTHERA.2013.10.011 24189487 PMC5098338

[B414] Woller S. A. Malik J. S. Aceves M. Hook M. A. (2014). Morphine Self-Administration following Spinal Cord Injury. *J. Neurotrauma* 31 1570–1583. 24827476 10.1089/neu.2013.3293 PMC4161170

[B415] Worley M. J. Heinzerling K. G. Shoptaw S. Ling W. (2015). Pain volatility and prescription opioid addiction treatment outcomes in patients with chronic pain. *Exp. Clin. Psychopharmacol.* 23 428–435. 10.1037/PHA0000039 26302337 PMC4658240

[B416] Worley M. J. Heinzerling K. G. Shoptaw S. Ling W. (2017). Volatility and change in chronic pain severity predict outcomes of treatment for prescription opioid addiction. *Addiction* 112 1202–1209. 10.1111/add.13782 28164407 PMC5461207

[B417] Xu M. Petraschka M. McLaughlin J. P. Westenbroek R. E. Caron M. G. Lefkowitz R. J. (2004). Neuropathic pain activates the endogenous kappa opioid system in mouse spinal cord and induces opioid receptor tolerance. *J. Neurosci.* 24 4576–4584. 10.1523/JNEUROSCI.5552-03.2004 15140929 PMC2376823

[B418] Xu X. J. Hao J. X. Wiesenfeld-Hallin Z. (1996). Nociceptin or antinociceptin: potent spinal antinociceptive effect of orphanin FQ/nociceptin in the rat. *Neuroreport* 7 2092–2094. 8930965

[B419] Yamamoto T. Nozaki-Taguchi N. Kimura S. (1997). Analgesic effect of intrathecally administered nociceptin, an opioid receptor-like receptor agonist, in the rat formalin test. *Neuroscience* 81 249–254. 9300417 10.1016/s0306-4522(97)00166-8

[B420] Yasuda K. Raynor K. Kong H. Breder C. Takeda J. Reisine T. (1993). Cloning and functional comparison of kappa and delta opioid receptors from mouse brain. *Proc. Natl. Acad. Sci. U.S.A.* 90 6736–6740. 8393575 10.1073/pnas.90.14.6736 PMC47007

[B421] Yovell Y. Bar G. Mashiah M. Baruch Y. Briskman I. Asherov J. (2016). Ultra-low-dose buprenorphine as a time-limited treatment for severe suicidal ideation: a randomized controlled trial. *Am. J. Psychiatry* 173 491–498. 10.1176/APPI.AJP.2015.15040535 26684923

[B422] Zajacova A. Grol-Prokopczyk H. Zimmer Z. (2021). Pain trends among american adults, 2002–2018: patterns, disparities, and correlates. *Demography* 58 711–738. 10.1215/00703370-8977691 33834222 PMC8035485

[B423] Zaveri N. T. Marquez P. V. Meyer M. E. Hamid A. Lutfy K. (2018). The nociceptin receptor (NOP) agonist AT-312 blocks acquisition of morphine- and cocaine-induced conditioned place preference in mice. *Front. Psychiatry* 9:638. 10.3389/fpsyt.2018.00638 30555362 PMC6281746

[B424] Zernig G. Ahmed S. H. Cardinal R. N. Morgan D. Acquas E. Foltin R. W. (2007). Explaining the escalation of drug use in substance dependence: models and appropriate animal laboratory tests. *Pharmacology* 80 65–119. 10.1159/000103923 17570954

[B425] Zhang G. Lagrange A. H. Rønnekleiv O. K. Kelly M. J. (1996). Tolerance of hypothalamic β-endorphin neurons to μ-opioid receptor activation after chronic morphine. *J. Pharmacol. Exp. Ther.* 277 551–558. 8613967

[B426] Zhang H. Kranzler H. R. Yang B. Z. Luo X. Gelernter J. (2008). The OPRD1 and OPRK1 loci in alcohol or drug dependence: OPRD1 variation modulates substance dependence risk. *Mol. Psychiatry* 13 531–543. 10.1038/SJ.MP.4002035 17622222 PMC3163084

[B427] Zhang X. Bao L. Shi T. J. Ju G. Elde R. Hökfelt T. (1998). Down-regulation of mu-opioid receptors in rat and monkey dorsal root ganglion neurons and spinal cord after peripheral axotomy. *Neuroscience* 82 223–240. 10.1016/S0306-4522(97)00240-6 9483516

[B428] Zhang X. Y. Dou Y. N. Yuan L. Li Q. Zhu Y. J. Wang M. (2020). Different neuronal populations mediate inflammatory pain analgesia by exogenous and endogenous opioids. *eLife* 9:e55289. 10.7554/eLife.55289 32519950 PMC7311172

[B429] Zhou J. Ma R. Jin Y. Fang J. Du J. Shao X. (2021). Molecular mechanisms of opioid tolerance: from opioid receptors to inflammatory mediators (Review). *Exp. Ther. Med.* 22 1–8. 10.3892/ETM.2021.10437 34345286 PMC8311239

[B430] Zhu Y. King M. A. Schuller A. G. P. Nitsche J. F. Reidl M. Elde R. P. (1999). Retention of supraspinal delta-like analgesia and loss of morphine tolerance in delta opioid receptor knockout mice. *Neuron* 24 243–252. 10.1016/S0896-6273(00)80836-3 10677041

[B431] Zöllner C. Stein C. (2007). Opioids. *Handb. Exp. Pharmacol.* 177 31–63. 10.1007/978-3-540-33823-9_2 17087119

[B432] Zubieta J. K. Smith Y. R. Bueller J. A. Xu Y. Kilbourn M. R. Jewett D. M. (2001). Regional Mu opioid receptor regulation of sensory and affective dimensions of pain. *Science* 293 311–315. 10.1126/science.1060952 11452128

[B433] Zubieta J. K. Smith Y. R. Bueller J. A. Xu Y. Kilbourn M. R. Jewett D. M. (2002). μ-opioid receptor-mediated antinociceptive responses differ in men and women. *J. Neurosci.* 22 5100–5107. 10.1523/jneurosci.22-12-05100.2002 12077205 PMC6757760

